# Multiomics Analysis Reveals that *GLS* and *GLS2* Differentially Modulate the Clinical Outcomes of Cancer

**DOI:** 10.3390/jcm8030355

**Published:** 2019-03-13

**Authors:** Subbroto Kumar Saha, S.M. Riazul Islam, M. Abdullah-AL-Wadud, Saiful Islam, Farman Ali, Kyoung Sik Park

**Affiliations:** 1 Department of Stem Cell and Regenerative Biotechnology, Konkuk University, 120 Neungdong-Ro, Seoul 05029, Korea; 2 Department of Computer Science and Engineering, Sejong University, 209 Neungdong-ro, Gwangjin-gu, Seoul 05006, Korea; riaz@sejong.ac.kr; 3 Department of Software Engineering, King Saud University, Riyadh 11543, Saudi Arabia; mwadud@ksu.edu.sa; 4 Department of Computer Science, King Saud University, Riyadh 11543, Saudi Arabia; saislam@ksu.edu.sa; 5 Department of Information and Communication Engineering, Inha University, Incheon 22212, Korea; farmankanju@gmail.com; 6 Department of Surgery, Konkuk University Medical Centre, 120 Neungdong-ro, Gwangjin-gu, Seoul 05029, Korea; 20090117@kuh.ac.kr

**Keywords:** glutaminase, *GLS*, *GLS2*, multiomics, prognostic biomarkers, clinical outcomes, cancer therapy

## Abstract

Kidney-type glutaminase (*GLS*) and liver-type glutaminase (*GLS2*) are dysregulated in many cancers, making them appealing targets for cancer therapy. However, their use as prognostic biomarkers is controversial and remains an active area of cancer research. Here, we performed a systematic multiomic analysis to determine whether glutaminases function as prognostic biomarkers in human cancers. Glutaminase expression and methylation status were assessed and their prominent functional protein partners and correlated genes were identified using various web-based bioinformatics tools. The cross-cancer relationship of glutaminases with mutations and copy number alterations was also investigated. Gene ontology (GO) and pathway analysis were performed to assess the integrated effect of glutaminases and their correlated genes on various cancers. Subsequently, the prognostic roles of *GLS* and *GLS2* in human cancers were mined using univariate and multivariate survival analyses. *GLS* was frequently over-expressed in breast, esophagus, head-and-neck, and blood cancers, and was associated with a poor prognosis, whereas *GLS2* overexpression implied poor overall survival in colon, blood, ovarian, and thymoma cancers. *Both* *GLS* and *GLS2* play oncogenic and anti-oncogenic roles depending on the type of cancer. The varying prognostic characteristics of glutaminases suggest that *GLS* and *GLS2* expression differentially modulate the clinical outcomes of cancers.

## 1. Introduction

Cancer is currently one of the leading causes of human death worldwide. Moreover, the global occurrence of cancer has been steadily growing over recent decades [1]. Although the overall diagnosis and treatment of cancers have improved, prognosis is still below standard. Therefore, the development of more effective biomarkers for prognosis is a highly desirable outcome of cancer research worldwide.

One of the salient features of cancer cells is that they have an altered metabolic activity compared with most normal tissue cells [2]. These alterations in cellular metabolism pathways are collectively called cancer metabolism and support the acquisition and maintenance of the malignant properties of cancer cells [3]. Of the numerous metabolic alterations, altered enzyme expression is a common phenomenon, and the expression of metabolic genes is often altered in cancer [4]. The amplification or deletion of genes, or epigenetic changes, are a few of the factors that result in deviations in metabolic enzyme expression [5]. Underlying mechanisms aside, modifications in basal enzymatic activity result in potential susceptibilities that could be targeted in cancer treatment. Glutaminase is an example of such an enzyme, the activity of which is noticeably dysregulated in several cancer cells [6]. This enzyme generates glutamate from glutamine. It is now recognized that the dysregulation of glutamine metabolism is a prominent phenomenon that results in the proliferation of cancer cells [7]. Glutamine, and hence glutaminase, have become appealing targets for cancer therapy. In humans, the glutaminase family consists of two key members—the kidney-type glutaminase (*GLS*, also improperly known as *GLS1*) and liver-type glutaminase (*GLS2*) genes—that encode several respective tissue-specific isoenzymes. Some studies have shown that *GLS* is regulated by oncogenes and largely contributes to tumor cell growth [8,9]. On the contrary, *GLS2* has tumor suppressant properties and places a limitation on malignancy [10,11]. Therefore, these glutaminase members, henceforth referred to as glutaminases, may be used as biomarkers for patient prognosis. Glutaminase-targeted therapy has been found to be effective in various clinical attempts [5,12,13]. This study investigates the prognostic significance of *GLS* and *GLS2* in human cancers using systematic bioinformatic analysis.

*GLS* has recently attracted attention as a biomarker for the prediction of prognosis in cancers, although its implications in cancer biology remain an area of active investigation. In contrast, studies on *GLS2* expression in cancer are relatively limited. Experimental trials have demonstrated that glutaminase-based molecular targeted therapy may lead to cancer cell apoptosis, thus preventing tumor development. However, the application of *GLS* and *GLS2*-mediated treatment in a large-scale clinical setting is still in its infancy, and the use of their expression levels as prognostic markers of human cancers is controversial. In this context, we evaluated the significance of glutaminase expression in various human cancers using a number of online bioinformatics platforms and tools. We comprehensively investigated the expression patterns, functions, and prognostic values of *GLS* and *GLS2* in cancers by accessing and analyzing all currently available gene expression data. This systemic analysis eventually determined whether glutaminase expression can be used as a biomarker for the prognosis of human cancers.

## 2. Materials and Methods

### 2.1. Transcript Expression Analysis Using Oncomine Platform

The expression levels of glutaminases in various cancers were retrieved from the Oncomine platform (https://www.oncomine.org/resource/login.html) [14,15]. This platform comes with a large collection of independent data sets and well curated data. Using the default settings for various groups of filters, such as primary filters, sample filters, and data set filters, each query for accessing mRNA expression data was executed using threshold parameters of *p*-value 1e-4, fold-change 2, and gene ranking in the top 10%. The precise analyses are summarized in Supplementary Tables S1 and S2.

### 2.2. Transcript Expression Analysis Using GENT 

Gene expression across normal and tumor tissue (GENT) (http://medical-genome.kribb.re.kr/GENT/) [16] is an online database which provides gene expression patterns across a range of human cancer and normal tissues. Using the default settings on data sets, samples, and probes, the query was performed for *GLS* and *GLS2* to obtain their respective U133Plus2 differential gene expression patterns in normal vs. cancer tissues. GENT-based glutaminase expression results were used to determine the differential gene expression pattern in reference to the Oncomine-based results.

### 2.3. Transcript Expression Analysis Using GEPIA 

The gene expression profiling interactive analysis (GEPIA) (http://gepia.cancer-pku.cn/) is another interactive online platform for analyzing RNA sequencing expression [17]. The GEPIA performs data mining based on TCGA data. The GEPIA-provided box plots tool was used for performing tumor/normal differential expression analysis for *GLS* and *GLS2* in different cancers. Similar to GENT, the glutaminases expression results obtained from GEPIA were also mainly used to crosscheck the results from the Oncomine platform. 

### 2.4. Methylation Status Analysis Using TCGA Wanderer 

TCGA Wanderer (http://maplab.imppc.org/wanderer/) is an online tool that performs data mining on TCGA data to interpret DNA methylation and gene expression data in human cancers [18]. This study utilized Wanderer to retrieve level 3 TCGA data for methylation arrays and expression through the portal’s gene cantered interactive web viewer. The imported data was then used to identify the statistically significant values of methylation levels of *GLS* and *GLS2* promoters in different cancers using GraphPad Prism 7 software using the unpaired *t*-test.

### 2.5. PPI Analysis Using STRING 

The search tool for the retrieval of interacting genes/proteins STRING (https://string-db.org/) is a web-based tool that provides a critical assessment and integration of protein-protein interactions [19]. In this study, STRING was applied to determine the functional protein partners of each glutaminase member. Text mining, experiments, databases, neighborhood, gene fusion, and co-expression were taken into consideration as active protein-protein interaction sources. The protein-protein network was constructed using both known and prediction interactions with no network clustering.

### 2.6. Analysis of Gene Expression and Mutation Alterations Using cBioPortal 

The cBioPortal for cancer genomics (http://www.cbioportal.org) is a recognized and widely used online portal that provides visualization and analysis of large-scale cancer genomics data sets [20,21]. In this study, cBioPortal was applied to analyze expression patterns, mutations, and CNAs of *GLS* and *GLS2* with appropriate parameter settings. In addition, the OncoPrint sub-tool of CBioPortal was used to analyze the integrated status of mutations and CNAs for glutaminases and their functional protein partners.

### 2.7. Survival Analysis Using Kaplan-Meier Plotter 

The Kaplan-Meier plotter (http://kmplot.com/analysis/) [22] is a web-based tool used to analyze the impact of various genes on the survival of cancer patients. In this study, the correlations between the expression of glutaminases and the survival of patients with cancers of interest were analyzed using the Kaplan-Meier plotter.

### 2.8. Survival Analysis Using PROGgeneV2 

The PROGgeneV2-Pan cancer prognostics database (http://watson.compbio.iupui.edu/chirayu/proggene/database/?url=proggene) is a web-based tool for survival analysis using gene expression [23]. In this study, PROGgeneV2 was applied to perform survival analysis on *GLS* and *GLS2* as a signature in various human cancers with OS as a survival measure and median bifurcate gene expression. Thus, the prognostic significance of the co-occurrence (high/high) and non-co-occurrence (low/low) of *GLS*/*GLS2* were evaluated.

### 2.9. Prognosis Analysis Using PrognoScan 

PrognoScan (http://dna00.bio.kyutech.ac.jp/PrognoScan/) [24] is an online platform that is used for the meta-analysis of the prognostic value of various genes. In this study, PrognoScan was used to assess the correlation between the expression of glutaminases and survival in several types of cancer. It was also applied to analyze the association of *GLS* and *GLS2* expression with the survival in various cancer patients. These results are briefly presented in Supplementary Tables S3 and S4.

### 2.10. Prognosis Analysis Using SurvExpress

SurvExpress (http://bioinformatica.mty.itesm.mx/SurvExpress) is a cancer-wide gene expression database with clinical outcomes and a web-based tool for survival analysis [25]. This database contains more than 39,000 samples and 225 datasets covering tumors in more than 26 different tissues. Using this platform, survival plots were generated for the functional protein partners of *GLS* and *GLS2* in specific cancer types using TCGA data. 

### 2.11. Survival and Correlation Analysis Using R2 

R2 (http://r2platform.com) is a versatile online platform for use in various genomics analysis and visualization [26,27]. In this study, R2 was applied to analyze the correlation between glutaminase expression and the survival of patients with certain cancers. The R2 software was also used to identify and import the list of genes correlated with *GLS* and *GLS2*. The imported data was then exploited to determine the glutaminase-correlated genes common to all cancers of interest using the functional enrichment analysis tool (FunRich) (http://www.funrich.org/) [28]. Then, the protein analysis through evolutionary relationships (PANTHER) (http://pantherdb.org/) [29] classification system was used to perform GO and pathway analyses of commonly correlated genes.

### 2.12. Statistical Analysis

Expression data were extracted from the Oncomine, cBioPortal, GEPIA, and GNET databases. The *p*-values < 0.05 were considered significant. Methylation data were extracted from TCGA Wanderer and used to generate box plots. The unpaired *t*-test was used to determine the *p*-values, where *p* < 0.05 was considered significant (* *p* < 0.05, ** *p* < 0.01, *** *p* < 0.001, and **** *p* < 0.0001). Survival curves were extracted from the R2: Kaplan Meier Scanner, PrognoScan, Kaplan-Meier Plotter, and PROGgeneV2 databases. All survival results are displayed with *p*-values obtained using the log-rank test. For multivariate survival analysis, the clinical outcome data were extracted from the PROGgeneV2 database. Moreover, clinical outcome data were retrieved from the Kaplan-Meier Plotter database. The data were then processed to generate multivariate survival curves using GraphPad Prism version 7. Log-rank *p*-values < 0.05 were considered significant, and *p* = *ns* was used to denote “not significant.”

## 3. Results

**Figure 1:**
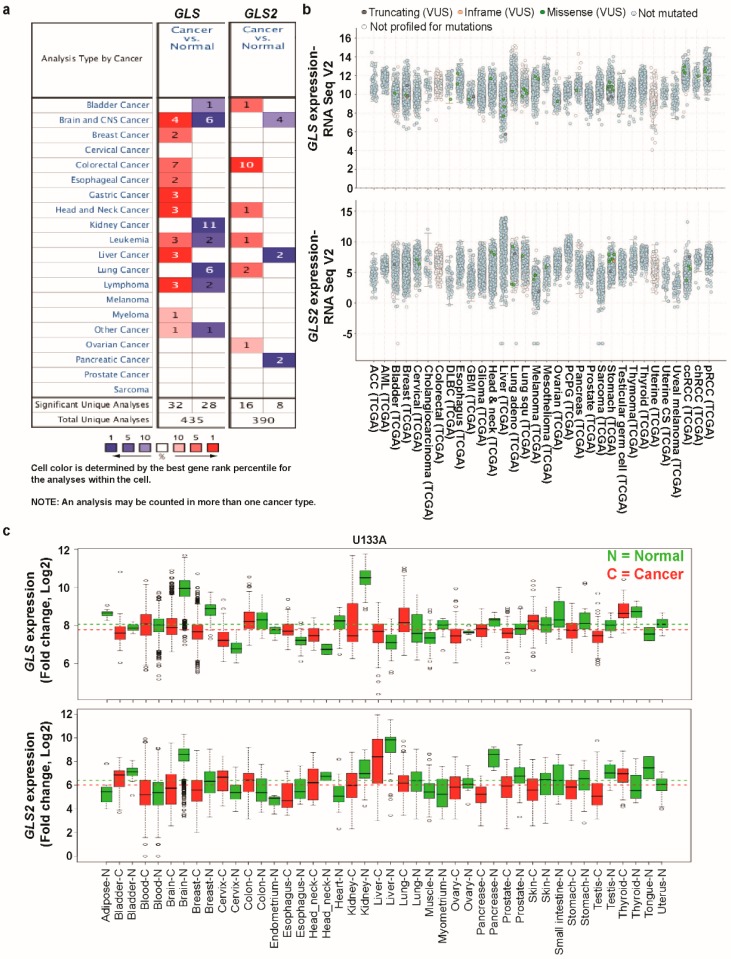
Transcription levels of GLS and GLS2 in different types of cancer (Oncomine, TCGA, and GENT databases). (**a**) This graphic was generated from the Oncomine database (available at https://www.oncomine.org/resource/login.html), indicating the number of data sets with statistically significant (*p* < 0.01) mRNA over-expression (red) or under-expression (blue) of *GLS* and *GLS2* (different types of cancer vs. corresponding normal tissue). The threshold was designed with following parameters: *p*-value of 1e-4, fold change of 2, and gene ranking of 10%. (**b**) The expression of *GLS* and *GLS2* in 32 types of human cancer was retrieved from the cBioPortal database (http://www.cbioportal.org/index.do). Every spot represents a single study. (**c**) Expression pattern of *GLS* and *GLS2* mRNA in normal and tumor tissues. GLS and GLS2 mRNA expression in various types of cancer was searched in the GENT database (available at http://medical-genomics.kribb.re.kr/GENT/). Boxes represent the median and the 25th and 75th percentiles; dots represent outliers. Red boxes represent tumor tissues; green boxes represent normal tissues. Red and green dashed lines represent the average value of all tumor and normal tissues, respectively.

We used the Oncomine database to determine the role of glutaminases in the development of human cancers. The investigation started with the evaluation of their transcription levels in cancers compared with that in normal tissues. Using the default filter settings, we used 1e-4, 2, and 10% as the *p*-value, fold change, and gene ranking, respectively. Using these threshold parameters, we queried the Oncomine database and found results on its visualization interface. Compared with their expression levels in normal tissues, both glutaminase members were highly upregulated in certain cancers and upregulated to a lesser extent in others. Based on these data-driven results, one may argue that the glutaminases play either an oncogenic or anti-oncogenic role, depending on the type of cancer (Figure 1a). A platform that contains a large collection of RNA sequencing studies is the cancer genome atlas (TCGA) database, which helps us understand the molecular basis of cancer. Another standard online bioinformatics platform is gene expression across normal and tumor tissue (GENT), which also provides the expression patterns of genes across a wide array of cancer and normal tissues. We accessed the TCGA data via the cBioPortal web and GENT database directly and examined the mRNA levels of glutaminases in various types of cancer. The results show that *GLS* and *GLS2* are differentially expressed (Figure 1b,c) in both the TCGA and GENT databases. Also, as we individually performed an average of the expression of glutaminases over the entire set of tissues, the average expressions of both *GLS* and *GLS2* were slightly lower in cancer tissues than in the normal tissues, as analyzed using the GENT database. The detailed analyses of glutaminases are presented below (Figure 1c).

### 3.1. Transcript Expression Analysis of Glutaminases

**Figure 2:**
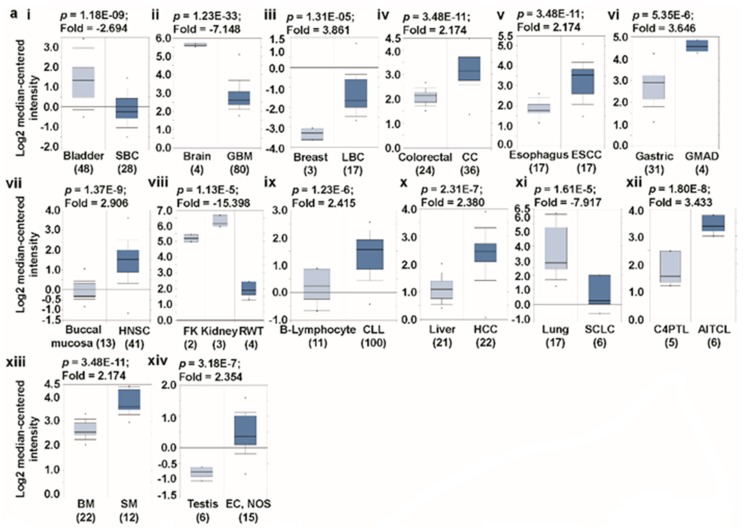

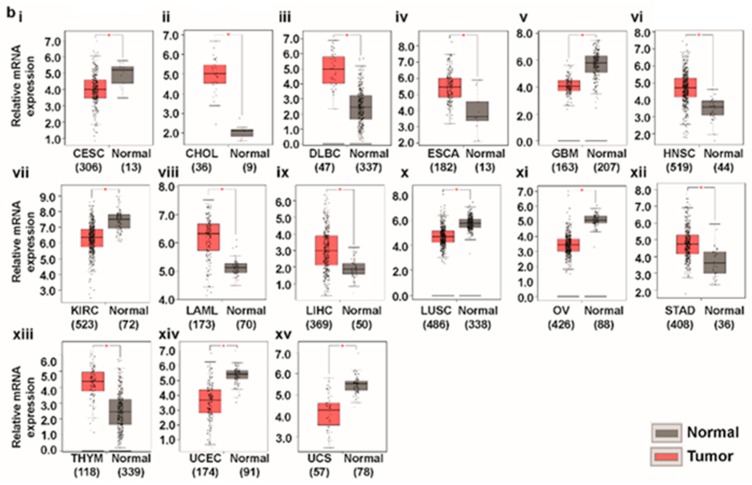
*GLS* expression analysis in different types of cancer (Oncomine and TCGA databases). (**a**) The box plot comparing specific *GLS* expression in normal (left plot) and cancer tissue (right plot) was derived from the Oncomine database. The fold change of *GLS* in various types of cancer was determined from our analyses as shown in Supplementary Table S1. The analysis was shown in SBC relative to normal bladder (i), GBM relative to normal brain (ii), LBC relative to normal breast (iii), CC relative to normal colorectal (iv), ESCC relative to normal esophagus (v), GMAD relative to normal gastric (vi), HNSC relative to normal buccal mucosa (vii), RWT relative to normal kidney (viii), CLL relative to B-lymphocyte (ix), HCC relative to normal liver (x), SCLC relative to normal lung (xi), AITCL relative to C4PTL (xii), SM relative to BM (xiii), and EC, NOS relative to normal testis (xiv). The threshold was designed using the following specific parameters: *p*-value = 1E-4, fold change = 2, and gene rank 10%. (**b**) The expression of the *GLS* gene in the Cancer Genome Atlas (TCGA) database. Box plots showing the *GLS* mRNA expression in various tumor (T) and normal (N) tissues, using data from the TCGA database via the GEPIA web (i–xv). The threshold was designed with the following specific parameters: *p*-value = 0.01, fold change = 2. (Abbreviations: SBC, bladder cancer (Sanchez-Carbayo Bladder 2); AOA, anaplastic oligo-astrocytoma (French Brain); GBM, glioblastoma (Murat Brain); LBC, lobular breast carcinoma (Zhao Breast); CC, colorectal carcinoma (Skrzypczak Colorectal); ESCC, esophageal squamous cell carcinoma (Hu Esophagus); GMAD, gastric mixed adenocarcinoma (DErrico Gastric); HNSC, head-and-neck squamous cell carcinoma (Ginos Head-Neck); FK, fetal kidney; RWT, renal Wilms tumor (Yusenko Renal); CCL, chronic lymphocytic leukemia (Haslinger Leukemia); C4PTL, CD4-positive T-lymphocyte; CATCL, chronic adult T-cell leukemia/lymphoma (Choi Leukemia); HCC, hepatocellular carcinoma (Roessler Liver); SCLC, small cell lung carcinoma (Bhattacharjee Lung); AITCL, angioimmunoblastic T-cell lymphoma (Piccaluga Lymphoma); BM, bone marrow; SM, smoldering myeloma (Zhan Myeloma 3); EC, NOS, embryonal carcinoma, NOS (Korkola Seminoma); CHOL, cholangiocarcinoma; DLBC, diffuse large B-cell lymphoma (Lymphoid Neoplasm); ESCA, esophageal carcinoma; GBM, glioblastoma multiforme; HNSC, head-and-neck squamous cell carcinoma; KIRC, kidney renal clear cell carcinoma; LAML, acute myeloid leukemia; LIHC, liver hepatocellular carcinoma; STAD, stomach adenocarcinoma; THYM, thymoma; UCEC, uterine corpus endometrial carcinoma; UCS, uterine carcinosarcoma.)

**Figure 3:**
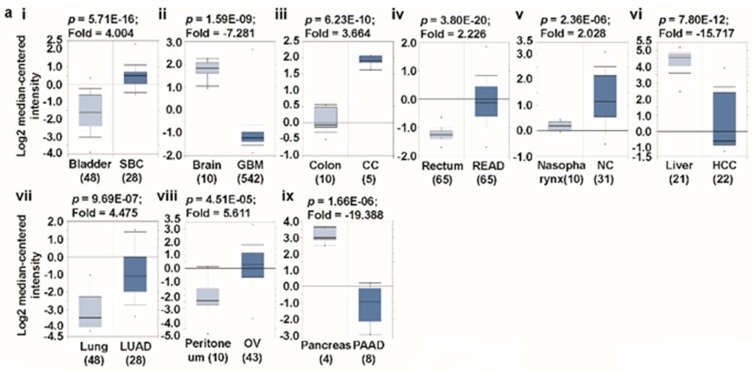

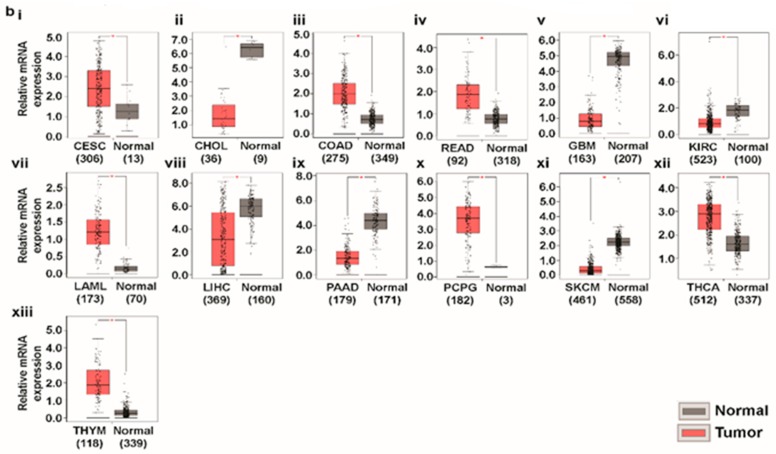
*GLS2* expression analysis in different types of cancer (Oncomine and TCGA databases). (**a**) The box plot comparing specific *GLS2* expression in normal (left plot) and cancer tissue (right plot) was derived from the Oncomine database. The fold change of *GLS2* in various types of cancer was identified from our analyses in Supplementary Table S2. The analysis was shown in SBC relative to normal bladder (i), GBM relative to normal brain (ii), CC relative to normal colorectal (iii), READ relative to normal rectum (iv), NC relative to normal nasopharynx (v), HCC relative to normal liver (vi), LUAD relative to normal lung (vii), OV relative to normal peritoneum (viii), and PAAD relative to normal pancreas (ix). The threshold was designed with the following specific parameters: *p*-value = 1e-4, fold change = 2, and gene rank 10%. (**b**) The expression of the *GLS2* gene in the Cancer Genome Atlas (TCGA) database. Box plots showing the GLS2 mRNA expression in various tumors (T) and normal (N) tissues, using data from the TCGA database via the GEPIA web (i-xiii). The threshold was designed with the following specific parameters: *p*-value = 0.01, fold change = 2. (Abbreviations: SBC, superficial bladder cancer (Sanchez-Carbayo Bladder 2); GBM, brain glioblastoma (TCGA); CC, colon carcinoma (Skrzypczak Colorectal 2); READ, rectal adenocarcinoma (Gaedcke Colorectal); NC, nasopharyngeal carcinoma; HCC, hepatocellular carcinoma (Roessler Liver); LUAD, lung adenocarcinoma (Stearman Lung); PAAD, pancreatic adenocarcinoma (Iacobuzio-Donahue Pancreas 2); CESC, cervical squamous cell carcinoma and endocervical adenocarcinoma; CHOL, cholangiocarcinoma; COAD, colon adenocarcinoma; READ, rectum adenocarcinoma; GBM, glioblastoma multiforme; KIRC, kidney renal clear cell carcinoma; LAML, acute myeloid leukemia; LIHC, liver hepatocellular carcinoma; PAAD, pancreatic adenocarcinoma; PCPG, pheochromocytoma and paraganglioma; SKCM, skin cutaneous melanoma; THCA, thyroid carcinoma; THYM, thymoma).

Using the differential analysis tool of the Oncomine database, we conducted a cDNA microarray analysis to investigate the expression patterns of glutaminases in various types of cancer. The database was successively queried for *GLS* and *GLS2* expression in all the cancers of interest and their respective normal tissues. The analysis demonstrates that *GLS* is over-expressed in breast, colorectal, esophagus, gastric, head-and-neck, B cells, piccaluga lymphoma, bone marrow, testis, and liver cancers, but is under-expressed in brain, bladder, kidney, and lung cancers than in the normal tissue (Figure 2a(i–xiii)) [30,31,32,33,34,35,36,37,38,39,40,41,42,43]. In order to validate our analysis of *GLS* expression using the Oncomine web, we conducted single gene analysis on this gene in GEPIA, another TCGA database-driven web portal for gene expression profiling and interactive analysis. The result presented in Figure 2b(i–xv) confirms *GLS* overexpression in esophagus, head-and-neck, B cells, and liver cancers, and under-expression in brain, lung, and kidney cancers. The expression patterns of *GLS2* in different types of cancer are substantially different from that of GLS. We noticed that compared with the normal tissues, *GLS2* is considerably over-expressed in bladder, colon, rectum, head-and-neck, peritoneum, and lung cancers, whereas it is under-expressed in brain, liver, and pancreatic cancer (Figure 3a(i–ix)) [30,33,39,44,45,46,47,48], according to the Oncomine-aided gene expression investigation. The above expression pattern of *GLS2* in colon, rectum, brain, and liver cancer has also been found via GEPIA-based gene expression analysis (Figure 3b(i–xiii)). It is worth mentioning that our results on *GLS* and *GLS2* expression are in agreement with those of previous studies. For example, whereas the over-expression pattern of *GLS* was observed in breast, liver, and colon cancer [49,50,51,52], the under-expression pattern of this gene was noted in bladder and lung cancers [53,54]. In contrast, our results showed that *GLS2* is over-expressed in bladder and lung cancers [55,56], whereas it is downregulated in liver cancer [57] which indicates that glutaminases are indeed differentially expressed in different types of human cancer.

### 3.2. The Promoter Methylation Status of Glutaminases in Different Types of Cancer

**Figure 4:**
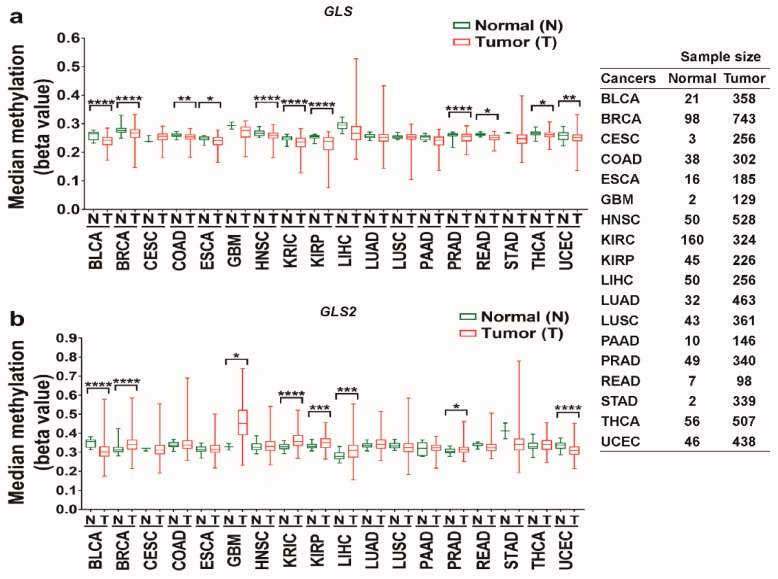
Promoter methylation levels of the *GLS* and *GLS2* genes in various types of cancer (TCGA Wanderer web). (**a**) Median methylation levels of *GLS* gene promoter in various types of cancer. (**b**) Median methylation levels of *GLS2* gene promoter in various types of cancer. The *p*-value was determined using unpaired *t*-test using GraphPad Prism 7 software. (*p*: * < 0.05, ** < 0.01, *** < 0.001, **** < 0.0001.) (Abbreviations: BLCA, bladder urothelial carcinoma; BRCA, breast invasive carcinoma; CESC, cervical squamous cell carcinoma; COAD, colon adenocarcinoma; ESCA, esophageal carcinoma; GBM, glioblastoma multiforme; HNSC, head-and-neck squamous cell carcinoma; KIRC, kidney renal clear cell carcinoma; KIRP, kidney renal papillary cell carcinoma; LIHC, liver hepatocellular carcinoma; LUAD, lung adenocarcinoma; LUSC, lung squamous cell carcinoma; PAAD, pancreatic adenocarcinoma; PRAD, prostate adenocarcinoma; READ, rectal adenocarcinoma; STAD, stomach adenocarcinoma; THCA, thyroid carcinoma; UCEC, uterine corpus endometrial carcinoma).

Methylation, an organized modification of protein and nucleic acid, was studied in the prognostic evaluation of different types of cancer, including liver, cervical and ovarian cancer [58,59,60]. The impact of the methylation of glutaminase promoter on certain types of cancer has been previously examined [57,61]. In this study, we investigated the methylation status of the promoters of the *GLS* and *GLS2* genes using TCGA Wanderer, an interactive web viewer for the visualization of DNA methylation based on TCGA data. The results showed that the promoter of the *GLS* gene is significantly hypomethylated in BLCA, BRCA, COAD, ESCA, HNSC, KIRC, KIRP, PRAD, READ, THCA, and UCEC, but it is not statistically significantly hypermethylated in any cancer tissue (Figure 4a). For the *GLS2* gene, it is hypomethylated in BLCA and UCAC, but hypermethylated in BRCA, GBM, KIRC, KIRP, and PRAD (Figure 4b). Interestingly, whereas the deficiency of the *GLS* gene promoter in ESCA and HNSC is consistent with the upregulation of *GLS* expression analyzed via GEPIA (Figure 2b(iv,vi)), the elevation of *GLS2* promoter in GBM, KIRC, and LIHC is in agreement with the downregulation of *GLS2* expression (Figure 3b(v,vi,viii)). In summary, the methylation statuses of glutaminases are differential in nature and could be used as biomarkers for certain cancers, such as ESCA, HNSC, GBM, KIRC, and LIHC.

### 3.3. Predicting Protein-Protein Interactions (PPIs) of Glutaminases

**Figure 5:**
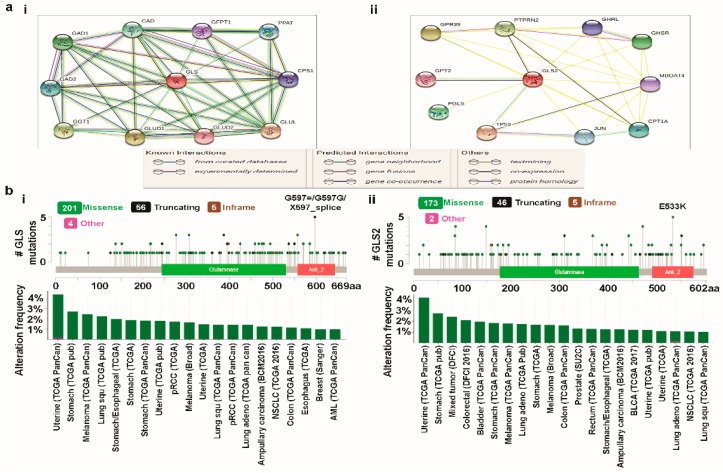

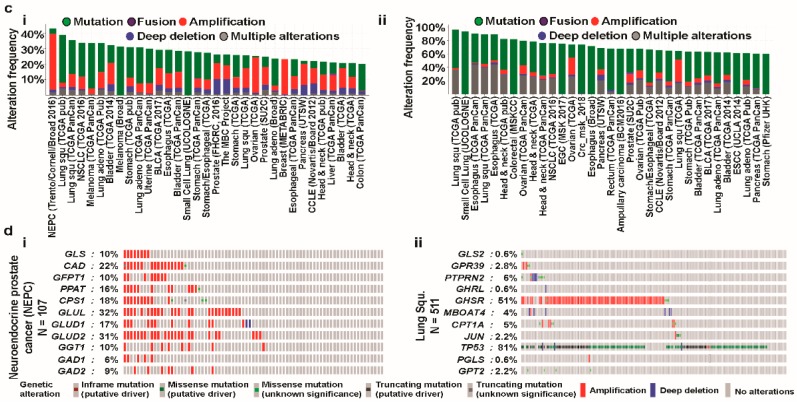
Identification of known and predicted structural proteins essential for *GLS* and *GLS2* function (STRING) and alteration frequency of mutations and copy number alterations (CNAs) in various types of cancer (cBioPortal web). (**a**) The interacting nodes are displayed in circles, obtained using Genemania. The predicted functional partners of *GLS* (i) and *GLS2* (ii) are shown, taking into consideration co-expression, co-localization, genetic interactions, pathway, physical interactions, predicted, and shared protein domains. (**b**) A total of 266 mutation sites were detected located between amino acids 0 and 669 of GLS protein. *GLS* mutations mainly occurred in uterine cancer and existed in a hotspot in the glutaminase domain (i). A total of 226 mutation sites were detected between amino acids 0 and 602 of GLS2 protein. GLS2 mutations mainly occurred in uterine cancer and existed in a hotspot in the N-terminal domain (ii). (**c**) The alteration frequency of an eleven-gene signature (*GLS, CAD, GFPT1, PPAT, CPS1, GLUL, GLUD1, GLUD2, GGT1, GAD1,* and *GAD2*) was determined using cBioPortal (http://www.cbioportal.org). Only the types of cancer containing >100 samples and an alteration frequency of >20% are shown. The alteration frequency included mutations (green), fusions (purple), amplifications (red), deep deletions (blue), or multiple alterations (grey) (i). The alteration frequency of an eleven-gene signature (GLS2, GPR39, PTPRN2, GHRL, GHSR, MBOAT4, CPT1A, JUN, TP53, PGLS, and GPT2) was determined using cBioPortal (http://www.cbioportal.org). Only the types of cancer containing >100 samples and an alteration frequency of >60% are shown. The alteration frequency included mutations (green), fusions (brawn), amplifications (red), deep deletions (blue), or multiple alterations (grey) (ii). (**d**) The *GLS* and *GLS2* gene-signatures were predominantly amplified and significantly co-expressed in neuroendocrine prostate cancer (NEPC) and ovarian cancer. We used the Oncoprint feature of cBioPortal (http://www.cbioportal.org) to determine the frequency of copy number alterations for each individual gene (*GLS, CAD, GFPT1, PPAT, CPS1, GLUL, GLUD1, GLUD2, GGT1, GAD1*, and *GAD2*) in GLS within the NEPC cancer subtypes. The alteration frequency included missense mutations (green), amplifications (red), deep deletions (blue), or no alterations (grey) (i). We used the Oncoprint feature of cBioPortal (http://www.cbioportal.org) to determine the frequency of copy number alterations for each individual gene (*GLS2, GPR39, PTPRN2, GHRL, GHSR, MBOAT4, CPT1A, JUN, TP53, PGLS,* and *GPT2*) in GLS2 within the ovarian cancer subtypes. The alteration frequency included missense mutations (green), amplifications (red), deep deletions (blue), or no alterations (grey) (ii).

*GLS* is located on human chromosome 2 and exists as different splice variants, namely, kidney glutaminase (KGA), glutaminase C (GAC), and GAM. This gene consists of 19 exons over 82 kb [56,62,63,64]. The N-termini of the *GLS* variants start with a 16-residue sequence, predicted to confine the proteins to the mitochondria [56]. *GLS2* is located on chromosome 12 and also appears as three transcriptional variants. Unlike *GLS* proteins, which differ in their C-termini, *GLS2* proteins differ at their N-termini. In their exons, *GLS* and *GLS2* exhibit high homology. *GLS* has, however, noticeably larger introns and untranslated regions of terminal exons. Although both glutaminase isozymes are activated by phosphate, *GLS2* is activated by a lower concentration of phosphate than *GLS* [65,66]. Whereas ammonia activates *GLS2*, *GLS* is inhibited by ammonia [67]. However, the underlying molecular mechanisms of these activation and inhibition process are largely unknown. Here, we tried to gain some insights at the system-level of the functional interactions of glutaminases with other closely related proteins via a protein–protein interaction (PPI) network. To identify the PPIs of glutaminases, we used STRING, an online biological database and web-based tool, to analyze known and predicted protein–protein interactions. The database was individually queried for *GLS* and *GLS2* with Homo sapiens as the organism, where the respective PPI network was obtained as the output (Figure 5a(i,ii)). Whereas each node in a PPI network represents all the proteins produced by a single, protein-coding gene locus, an edge represents protein-protein associations. An association, however, does not necessarily represent a physical binding between the pair of genes. Rather, associated proteins jointly contribute to a shared function. One can note that STRING performs text mining on a large number of scientific papers from various sources, such as PubMed, to search for statistically significant co-occurrences of gene names. We selected the prominent predicted functional protein partners of glutaminases from this analysis, as mentioned below, for further investigation. The predicted functional protein partners of GLS, along with their respective genes, are: carbamoyl-phosphate synthetase 2, aspartate transcarbamylase, dihydroorotase (CAD), glucosamine—fructose-6-phosphate aminotransferase isomerizing 1 (GFPT1), phosphoribosyl pyrophosphate amidotransferase (PPAT), carbamoyl-phosphate synthase 1 (CPS1), glutamate-ammonialigase (GLUL), glutamate dehydrogenase 1 (GLUD1), glutamate dehydrogenase 2 (GLUD2), gamma-glutamyltransferase 1 (GGT1), glutamate decarboxylase 1 (GAD1), and glutamate decarboxylase 1 (GAD2) (Figure 5a(i)). The predicted functional protein partners of *GLS2* along with their corresponding genes are: G protein-coupled receptor 39 (GPR39), protein tyrosine phosphatase, receptor type N2 (PTPRN2), ghrelin and obestatin prepropeptide (GHRL), growth hormone secretagogue receptor (GHSR), membrane bound O-acyltransferase domain containing 4 (MBOAT4), carnitine palmitoyltransferase 1A (CPT1A), jun proto-oncogene, AP-1 transcription factor Subunit (JUN), tumor protein P53 (TP53), 6-phosphogluconolactonase (PGLS), and glutamic-pyruvic transaminase 2 (GPT2) (Figure 5a(ii)).

It is worth noting that the STRING database is a curated database from the literature for PPI. On that, in addition to the STRING, we attempted to use the GeneMANIA. Nevertheless, the sets of proteins constituting the PPI networks using direct connections to the glutaminases were entirely different in these two databases with no common proteins/genes. The source of the contradiction in PPI results between STRING and GeneMANIA databases may be the inadequate number of studies and reports. Interestingly, the proteins constituting the PPI network found in STRING (also in GeneMANIA) were however indirectly connected to glutaminases when we performed the analysis in FunRich. In this study, although we selected the STRING to investigate the mutations and CNAs, we have provided the PPI networks obtained by FunRich analysis to show the indirect connections of the proteins found in STRING (and GeneMANIA) to GLS/GLS2 (Supplementary Figure S1). One can further note that it is intuitive that functional protein partners of glutaminases would experience various genetic alterations including mutations and CNAs, irrespective of the database.

### 3.4. Cross-Cancer Relationship of Glutaminases with Mutations and Copy Number Alterations (CNAs)

We individually examined the genetic alterations of *GLS* and *GLS2* in various types of cancer using cBioPortal and subsequently provided a comparative report with respect to the functional protein partners of glutaminases obtained from the PPI network analysis. Based on the entire set of available samples and studies, with clinical data from 69,758 patients, we queried the database for the *GLS* gene. Of the queried samples, 216 samples were associated with an altered gene set or pathways resulting in a somatic mutation frequency of 0.3%. As presented in Figure 5b(i), 266 mutations, including 201 missense, 56 truncating, and 5 inframe mutations, were detected. The mutations sites were located between amino acids 0 and 669. It is worth noting that, in the above mutation statistics, there exist at least 100 duplicate mutations in patients with multiple samples. We also observed that *GLS* mutation mainly occurred in uterine, lung, and stomach cancer and spans over the glutaminase (located between amino acids 244 and 530) and ankyrin repeat (located between amino acids 557 and 643) domains, with a hotspot in G597=/X597_splice/G597G, where 5 mutations were reported. Using the same settings as for *GLS*, we also queried the database for *GLS2*. In this case, we once again obtained 216 samples which were associated with an altered gene set or pathways resulting in the same somatic mutation frequency as *GLS*. However, the mutation statistics for *GLS2* were significantly different from those of *GLS*. In *GLS2*, there were 226 mutations in total, including 175 missense, 46 truncating, and 5 in-frame mutations. In addition, at least 75 duplicate mutations existed in patients with multiple samples. The mutations sites for *GLS2* were located between amino acids 0 and 602, and are thus, slightly denser than *GLS* in terms of their location. Like *GLS, GLS2* mutations also mainly occurred in uterine, lung, and stomach cancer, spanning over the glutaminase (located between amino acids 177 and 463) and Ankyrin repeats (located between amino acids 490 and 575) domains, with a hotspot at E533K (Figure 5b(ii)). We also provided a summary of the cancer types to gain a comparative understanding of cancer-wise alteration frequencies, where only cancers with at least 100 samples with at least 1% mutation were included. This summary shows that the maximum alteration occurs in uterine cancer for both *GLS* and *GLS2*.

Next, we investigated the integrated status of mutations and CNAs for *GLS* and its functional protein partners. The query was composed of both mutations and CNAs molecular profiles covering all the available cancer studies with the user defined gene list as *GLS, CAD, GFPT1, PPAT, CPS1, GLUL, GLUD1, GLUD2, GGT1, GAD1,* and GAD2. Then, we obtained the alteration frequency of this eleven-gene signature using 100% and 20% as a minimum number of total cases and a minimum percentage of altered cases, respectively (Figure 5c(i)). The results showed that the alteration frequency ranged from 25.27% to 52.34%. The alterations in the GLS-centered-signature-gene occur most dominantly in neuroendocrine prostate cancer (NEPC). Similarly, the analysis of the integrated status of mutations and CNAs for the *GLS2* gene and its functional protein partners (Figure 5c(ii)) resulted in alteration frequency ranges of 20.2% to 44.9%, with a threshold of 60% as a minimum percentage of altered cases. In this case, the query contained the user-defined gene list as *GLS2, GPR39, PTPRN2, GHRL, GHSR, MBOAT4, CPT1A, JUN, TP53, PGLS*, and *GPT2*. Unlike *GLS*, the alterations in the GLS2-centered-signature-gene occur mainly in lung cancer. Whereas the contribution of the GLS-centered-signature-gene to the alteration in NEPC largely results from amplification, the role of GLS2-centered-signature-gene on alteration in lung cancer appears in the form of mutations.

Next, we accessed the OncoPrint feature of cBioPortal to determine how genomic alterations in NEPC and lung cancer are disseminated over the aforementioned *GLS*-centered-signature-gene and GLS2-centered-signature-gene, respectively. Compared to *GLS2*-centered gene set, the fluctuation range of genetic alterations is significantly larger in the *GLS*-centered gene set (Figure 5d(i,ii)). Of the genes corresponding to the functional protein partners of GLS, GLUL and GLUD2 experience the most prominent genetic alterations, accounting for 32% and 31%, respectively (Figure 5d(i)). For the GLS2-centered-signature gene, the alterations largely take place in the TP53 and GHSR genes, accounting for 81% and 51%, respectively (Figure 5d(ii)). It is worth noting that whereas the alteration in GLUL solely occurred due to amplification, the alteration in TP53 largely takes place due to mutations. This result is consistent with the overall integrated status of mutations and CNAs for glutaminases and their functional protein partners, as explained in the preceding paragraph. As a consequence, the above cross-cancer analyses of glutaminases elucidated a differential role of glutaminases regarding mutations and CNAs.

### 3.5. Prognosis Estimation of Glutaminases

To understand how glutaminases influence the prognostic characteristics of different cancer patients, we examined the association between alterations in the expression of glutaminase-encoding genes and the clinical outcomes. The examination was conducted via several online genomics analysis platforms, namely, R2, PrognoScan, Kaplan-Meier Plotter, and the PROGgeneV2 database.

**Figure 6:**
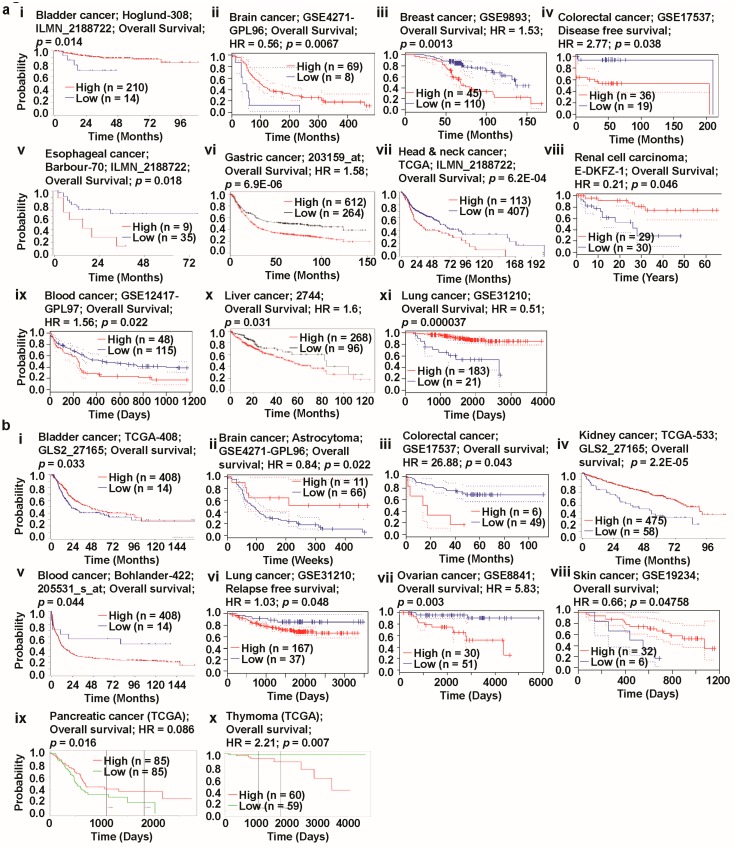
Correlation of *GLS* and *GLS2* gene expression with various cancer prognoses (R2: Kaplan Meier Scanner, PrognoScan, Kaplan-Meier Plotter, and PROGgeneV2 databases). (**a**) Survival curve comparing patients with high (red) and low (blue) expression of *GLS*, plotted using the R2 database for bladder (i), esophageal (v), and head-and-neck (vii) cancer; survival curve comparing patients with high (red) and low (black) expression of GLS, plotted using the PrognoScan database for brain (ii), breast (iii), colorectal (iv), kidney (viii), blood (ix), and lung (xi) cancer; survival curve comparing patient with high (red) and low (black) expression of *GLS*, plotted using the Kaplan-Meier Plotter database for gastric (vi) and liver (x) cancer. The threshold of Cox *p*-value < 0.05. (**b**) Survival curve comparing patients with high (red) and low (blue) expression of *GLS2*, plotted using the R2: Kaplan Meier Scanner database in bladder (i), kidney (iv), and blood (v) cancer; survival curve comparing patients with high (red) and low (blue) expression of *GLS2*, plotted using the PrognoScan database for brain (ii), colorectal (iii), lung (vi), ovarian (vii), and skin (viii) cancer; survival curve comparing patients with high (red) and low (green) expression of *GLS2,* plotted using the PROGgeneV2 database for pancreatic (ix) and thymoma (x) cancer. The threshold of Cox *p*-value < 0.05.

We observed a positive correlation between GLS overexpression and poor patient survival in breast, esophagus, head-and-neck, and blood cancer (Figure 6a(iii,v,vii,ix)). In contrast, the low expression of this gene was positively correlated with high overall survival (OS) in breast, colon, and esophagus cancer (Figure 6a(iii,iv,v,)). Also, patients with low *GLS* expression exhibited a positive correlation with poor OS in bladder, kidney, and lung cancer (Figure 6a(i,viii,xi)). In these three cancers, the high expression of the first member of the glutaminase family was, however, associated with high survival, as presented in the same figures. The OS characteristics associated with *GLS* in brain, gastric, blood, and liver cancer were similar, where both high and low expressions were positively correlated with poor survival (Figure 6a(ii,vi,ix,x)). It is, however, worth mentioning that the degree of poor survival in this category was more severe in patients with brain cancer compared to those with other types of cancer (Figure 6a(ii)). Remarkably, the Oncomine database also showed both high and low expression levels of GLS in brain and blood cancer (Figure 1a), depending upon the samples and respective analyses. We found that the relationship between the expression pattern of *GLS2* and the clinical outcome of most types of cancer was substantially different for *GLS*. A significant positive correlation between GLS2 overexpression and poor OS was observed in patients with colon, blood, ovarian, and thymoma cancer (Figure 6b(iii,v,vii,x)). The underexpression of *GLS2* in these types of cancer was, however, positively correlated with high survival. The upregulation of *GLS2* was also positively correlated with high survival in cancers including brain, kidney, and skin cancer (Figure 6b(ii,iv,viii)). In these cancers, the decrease in expression levels of the second member of the glutaminase family, however, showed a positive correlation with low survival. Moreover, any deviation in *GLS2* expression, compared to the expression mark observed in normal tissues, was associated with poor prognosis in bladder and pancreatic cancer in general (Figure 6b(i,ix). In contrast, both the overexpression and underexpression of *GLS2* were positively correlated with high survival in lung cancer (Figure 6b(vi)). Although the survival analysis uncovered both high and low expression levels of *GLS2* in certain types of cancer, including bladder and lung cancer, the Oncomine databased was unable to provide such expression patterns (Figure 1a), unlike for *GLS*.

To obtain further insights into the prognostic characteristics associated with glutaminases, we investigated the prognostic values of *GLS* and *GLS2* expression levels for different types of cancer (Supplementary Tables S3 and S4) using the ProgonoScan database. This investigation found that *GLS* was associated with a very poor prognosis in patients with breast, blood, soft tissue, and ovarian cancer—the overexpression of *GLS* was highly positively correlated with low survival. Although we were unable to crosscheck the expression pattern of *GLS* in soft tissue cancer, due to a lack of data, the high expression levels of this gene were confirmed in breast and blood cancer (Figure 1a), according to the Oncomine platform. The poor prognosis in ovarian cancer patients with *GLS* expression was, however, not in agreement with the GENT database which showed the downregulation of this gene (Figure 1c). We also found that GLS2 expression was associated with an extremely poor prognosis in colon, ovarian, and breast cancer. Whereas the overexpression of this gene in the first two types of cancer was confirmed by the Oncomine web (Figure 1a), data on the expression pattern of the second member of the glutaminase family in breast cancer were not available neither on the Oncomine database nor on the GENT database.

In summary, our analyses of patient survival using different platforms, including R2, Oncomine, and PrognoScan, underlined the oncogenic role of *GLS* in breast and blood cancer. However, we obtained contradicting results for the role of *GLS* in ovarian cancer. In contrast, the oncogenic role of *GLS2* in colon and ovarian cancer suggested that different factors should be taken into account for cancer prognosis, to clarify the aforementioned differential prognostic characteristics of *GLS* and *GLS2*.

**Figure 7:**
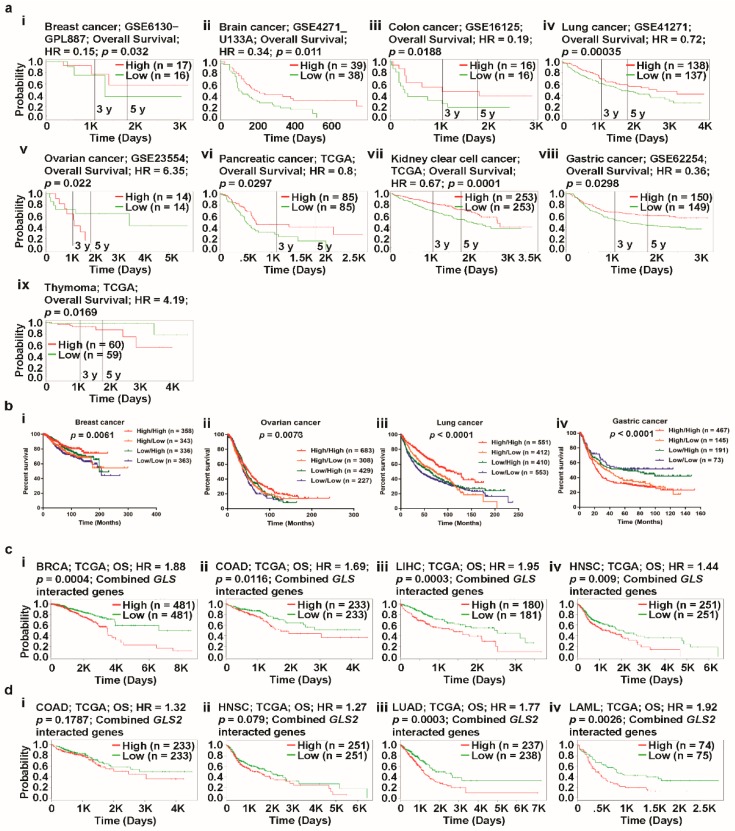
Correlation of combined *GLS* and *GLS2* gene expression with various cancer prognoses (PROGgeneV2 and Kaplan-Meier Plotter databases). (**a**) Survival curve comparing patients with high (red) and low (green) expression of combined *GLS* and *GLS2*, plotted using the PROGgeneV2 database in breast (i), brain (ii), colon (iii), lung (iv), ovarian (v), pancreatic (vi), kidney (vii), gastric (viii), and thymoma (ix) cancer; the cohort was divided according to the median of gene expression, with a threshold of Cox *p*-value < 0.05. (**b**) Survival curve comparing patients with high/high (red), high/low (orange), low/high (green), and low/low (blue) expression of combined *GLS* and *GLS2*, plotted using the data retrieved from Kaplan-Meier Plotter database for breast (i), ovarian (ii), lung (iii), and gastric (iv) cancer; with a threshold of Cox *p*-value < 0.05. (**c**) Survival curve comparing patients with high (red) and low (green) expression of combined *GLS* with 10 interacted genes from PPI data plotted using the SurvExpress database in BRCA (i), COAD (ii), LIHC (iii), HNSC (iv) cancer; the cohort was divided according to the median of gene expression, with a threshold of Cox *p*-value < 0.05. (**d**) Survival curve comparing patients with high (red) and low (green) expression of combined *GLS2* with 10 interacted genes from PPI data plotted using the SurvExpress database in COAD (i), HNSC (ii), LUAD (iii), LAML (iv) cancer; the cohort was divided according to the median of gene expression, with a threshold of Cox *p*-value < 0.05. (Abbreviations: BRCA, breast invasive carcinoma; COAD, colon adenocarcinoma; HNSC, head-and-neck squamous cell carcinoma; LIHC, liver hepatocellular carcinoma; LUAD, lung adenocarcinoma; LAML, Acute Myeloid Leukemia).

To investigate the combined impact of *GLS* and *GLS2* expression on the OS outcomes of patients with different types of cancer, we used the PROGgeneV2 online tool to study the prognostic associations of genes in various cancers. Using median gene expression values as bifurcation points, Cox proportional hazards regression analysis showed that patients with ovarian and thymoma cancer had considerably lower rates of OS with a worse prognosis when co-occurrence (high/high) of *GLS/GLS2* took place (Figure 7a(v)). In the case of other cancers of interest, such as breast, brain, and colon cancer, the non-co-occurrence of *GLS/GLS2* (low/low) is associated with a poor prognosis compared to that of co-occurrence (Figure 7a(i–iv,vi–viii)). Using the PROGgeneV2 biomarker identification tool, the impact of co-occurrence (high/high) and non-co-occurrence (low/low) of genes of interest in human cancers can be analyzed. In this online platform, however, the effect of partial co-occurrence (low/high or high/low) of a gene pair cannot be performed. As such, in order to gain some insights into the relationship between prognosis and partial-co-occurrence of *GLS/GLS2*, we retrieved clinical prognosis data of patients with breast, ovarian, lung, and gastric cancer from the Kaplan-Meier Plotter database. The clinical prognosis data were then used to prepare a multivariate survival plot to determine the co-occurrence characteristics of said gene pair. We noticed that whereas non co-occurrence and a partial co-occurrence (low/high) of *GLS/GLS2* showed a comparable prognosis in breast cancer (Figure 7a(i)), a partial-co-occurrence of *GLS*/*GLS2* did not provide any additional prognostic information in ovarian cancer (Figure 7a(ii)). Interestingly, multivariate survival analysis elucidated a significantly poorer prognosis in lung and gastric cancer when *GLS*/*GLS2* expression was low/high compared to co-occurrence (high/high) or non-co-occurrence (low/low) (Figure 7b(iii,iv)), suggesting that a partial-co-occurrence of *GLS/GLS2* may regulate cancer prognosis.

Next, we focused on the prognostic role of GLS and GLS2-centered-signature-gene set retrieved from PPIs network (see Figure 5a(i,ii)) in selected cancers with a high expression of GLS and *GLS2* using SurvExpress web. As the PPI partners regulate their expression and physiological role in cancer, it may obviously regulate the cancer prognosis. To find the prognostic relationship of *GLS* and *GLS2*-centered-signature-gene set in cancer, we performed survival analysis with all the retrieved PPI partners of *GLS* and *GLS2* using TCGA data. The combined *GLS*-centered-signature-gene set expression was significantly regulated with poor prognosis in breast, colon, liver, and head & neck cancers (See Figure 7c(i–iv)). On the other hand, the combined expression of GLS2-centered-signature-gene set was significantly regulated with poor prognosis in lung and leukemia while there was no significant relationship of clinical outcomes in colon and head & neck cancers (see Figure 7d(i–iv)), suggesting that interacting partners of *GLS* and *GLS2* together regulate the clinical outcomes in cancers. 

Taken together, multivariate survival analysis demonstrated that co-occurrence/partial occurrence/non co-occurrence of *GLS* and *GLS2,* combined *GLS* and *GLS2*-centered-signature-gene sets affected the clinical outcomes of patients with certain types of cancer. This data-driven result could help us to expand our understanding of the underlying molecular mechanisms of cancer prognosis with respect to glutaminase and its interacting partners’ expression.

### 3.6. Correlated Genes of Glutaminases and Their Functional Gene Ontology and Pathways

**Figure 8:**
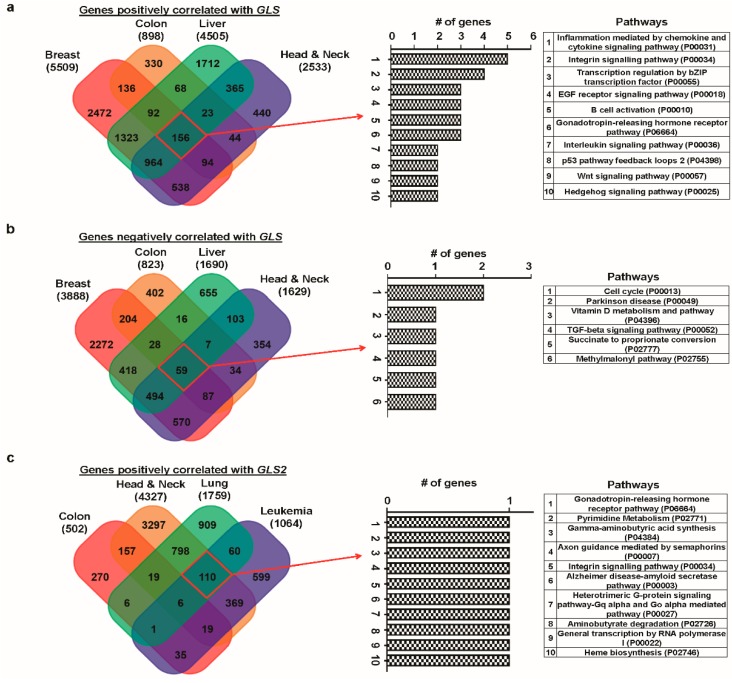

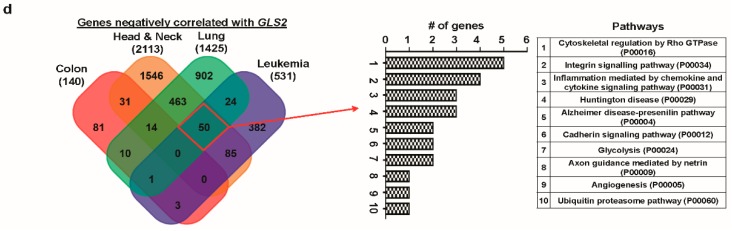
Analysis of positively and negatively correlated genes of *GLS* and *GLS2* using FunRich software and their predicted pathway analysis using PANTHER web. (**a**) Venn diagram of genes positively correlated to *GLS*, showing coincident genes in breast, colon, liver, and head-and-neck cancer; pathway analysis using PANTHER web and subsequently classified based on their pathway. (**b**) Venn diagram of genes negatively correlated to *GLS*, showing coincident genes in breast, colon, liver, and head-and-neck cancers; pathway analysis using PANTHER web and subsequently classified based on their pathway. (**c**) Venn diagram of genes positively correlated to *GLS2*, showing coincident genes in colon, head-and-neck, lung, and leukemia cancers; pathway analysis using PANTHER web and subsequently classified based on their pathway. (**d**) Venn diagram of genes negatively correlated to *GLS2*, showing coincident genes in colon, head-and-neck, lung, and leukemia cancers; pathway analysis using PANTHER web and subsequently classified based on their pathway.

A gene taking part in a signaling pathway is normally expressed with other genes, such that various genes collectively play a significant role in human cancer. Here, we identified genes that correlate with *GLS* and *GLS2* in certain selected cancers using the R2 platform. For each glutaminase member, we selected a set of the top four types of cancer based on the respective overexpression nature of that gene. Thus, we formed a cancer set A using breast, colon, liver, and head-and-neck cancer for *GLS*, whereas colon, head-and-neck, lung, and leukemia cancer formed the cancer set B for *GLS2*. We found 156 genes (hereafter referred to as “GLS-centered positive cluster”) correlated positively with *GLS* that were common in all the elements in cancer set A (Figure 8a). For *GLS2*, six positively correlated common genes (hereafter referred to as “*GLS2*-centered positive cluster”) were observed for cancer set B (Figure 8c). In addition, 59 negatively correlated common genes (hereafter referred to as “GLS-centered negative cluster”) were found for *GLS* in cancer set A (Figure 8b). In contrast, no negatively correlated common genes were found for *GLS2* in cancer set B (Figure 8d). The above correlation analysis indicated that *GLS* and *GLS2* show different correlation associations with other genes in the respective cancer set of interest. This suggests that each glutaminase member and their respective correlated genes participate in various common gene regulatory processes.

Next, we carried out pathway analysis and gene ontology (GO) for the aforementioned GLS/GLS2-centered positive/negative clusters using PANTHER, a web-based tool to classify proteins (and their genes), and Network Ontology Analysis (NOA), another online tool to analyze functions of a gene network. We noticed that although both *GLS*-centered positive and *GLS2*-centered positive clusters affected 10 pathways, only a few pathways were common between them (Figure 8a,c). Whereas inflammation mediated by the chemokine and cytokine signaling pathway and integrin signaling pathway are two major pathways in the GLS-centered positive cluster’s pathway, each of which are collectively regulated by at least four genes, each pathway contributed equally to the GLS2-centered positive cluster’s pathway. GLS-centered negative cluster was also found to regulate a relatively lower number of pathways when compared with its *GLS2* counterpart, indicating more diverse roles (Figure 8b,d).

At this point, we would like to draw attention to the possibility of eventually being able to derive signaling pathways based on PPI analysis. However, in our paper, the gene list (called correlated genes) attained from the analysis by using R2 is characteristically different from that (called functional protein partners) found from the PPI analysis. Whereas the former represents the co-expression, the latter symbolizes the physical network. In one hand, the correlated genes are the pairs of genes that show a similar expression pattern across samples, since the transcript levels of two co-expressed genes rise and fall together across samples. Gene co-expression networks are of biological interest since co-expressed genes are controlled by the same transcriptional regulatory program, functionally related, or members of the same pathway or protein complex. Conversely, PPIs are the physical contacts of high specificity established between two or more protein molecules as a result of biochemical events steered by electrostatic forces including the hydrophobic effect. On that, we have used the PPI and correlated gene lists for two entirely different purposes. Because a genetic alteration is inherently a physical manipulation, we used PPI to investigate the mutations and CNAs. In contrast, we used correlated genes to gain some insights into the signaling pathways.

We studied the functional classification of the correlated genes in the form of sub-ontologies, namely biological processes, cellular components, and molecular functions, for each GO. Considering a corrected *p*-value of less than 0.01, we found that although cellular macromolecule metabolic and RNA metabolic processes are two significant biological processes associated with *GLS*-centered positive cluster GO (Supplementary Table S5), branched chain family amino acid catabolic and branched chain family amino acid metabolic processes are the major biological processes corresponding to the *GLS2*-centered positive cluster GO (Supplementary Table S7). In addition, whereas the major component representing the molecular functions associated with the *GLS2*-centered positive cluster is acyl-CoA dehydrogenase activity, there is no statistically significant molecular function in relation to the *GLS*-centered positive cluster. There are several significant cellular components for *GLS2*-centered positive cluster GO. In contrast, the “nuclear part” and the “nucleus” are the only cellular components that make the cellular component sub-ontology for *GLS*-centered positive cluster. The GO results for *GLS*/*GLS2*-centered negative cluster are provided in Supplementary Tables S6 and S8. In summary, our results suggest that *GLS* and *GLS2* take part in different pathway regulatory processes and demonstrated the greatest differences in terms of correlations and functional behaviors in the signaling pathways.

## 4. Discussion

Several studies have showed that glutaminases contribute to the growth of various human cancers [7,49,68,69]. In addition, a number of studies have argued that *GLS* and *GLS2*-mediated targeted therapies are promising molecular medicines that inhibit cancer development [68,70,71]. Nevertheless, the impact of these glutaminase family members on the progress of cancers is still not well understood. To provide a systematic understanding regarding the role of *GLS* and *GLS2* in cancer prognosis, we performed comparative data mining on numerous gene expression data sets. GLS was found to be upregulated in breast, colorectal, esophagus, gastric, head-and-neck, B cell, piccaluga lymphoma, bone marrow, testis, and liver cancer, but downregulated in brain, bladder, kidney, and lung cancer according to the Oncomine-based expression analysis. In contrast, *GLS2* was upregulated in bladder, colon, rectum, head-and-neck, peritoneum, and lung cancer, but downregulated in brain, liver, and pancreatic cancer. The resulting gene expression patterns suggest that *GLS* and *GLS2* are differentially expressed in cancer versus normal tissues and that the magnitude of their expression also varies depending on the tissue type.

Next, we investigated the methylation status of the promoters of glutaminases to assess the extent of their impact on the corresponding gene expression and subsequently affect patient survival in cancer. In this agreement, previous studies demonstrated that DNA methylation is associated with cancer progression and prognosis by regulating gene stability and transcript [72,73,74]. The methylation level of the promoter of the *GLS* gene was found to be decreased in different types of cancer, including bladder, breast, colon, esophageal, head-and-neck, and kidney cancer. In contrast, *GLS2* methylation was found to be decreased in bladder cancer, but increased in breast, brain GBM, kidney, and prostate cancer. The resulting methylation statuses, which conform to the expression and clinical outcomes data, suggest that glutaminases could be labeled as biomarkers for certain types of cancer, including bladder, breast, esophageal, head-and-neck, kidney, and liver cancer.

CNAs are associated with a wide range of human cancers [75] and the area of this structural variation in the human genome can thus be utilized to develop molecular treatments for cancer. Previous studies commonly reported that mutations in oncogenes such as *KRAS*, *PIK3CA*, *BRAF* were associated with clinical outcomes in various cancers [76,77,78,79]. CNAs in oncogenes contribute important characteristics in clinical prognosis. Studies reported to be a link between CNAs and prognosis, in elaboration, diploid tumors were associated with better prognosis than the aneuploid tumors [80,81,82]. Later on, these reports largely studied either on arm-length changes in tumors or on CNAs in single oncogene or tumor suppressor [83,84,85,86,87]. However, the functional importance of CNAs and mutations for single gene in clinical prognosis remain unknown. As such, next, we determine which types of cancer were connected with significant CNAs in the glutaminase-gene signature using cBioPortal. *GLS* mutations mainly occurred in uterine, lung, and stomach cancer, whereas *GLS*2 mutations were mainly found in uterine, lung, and stomach cancer. We also examined the combined status of mutations and CNAs of each glutaminase member and their respective functional protein partners, identified using STRING-based PPI network analysis. For *GLS*, the genetic alterations of genes corresponding to its functional protein partners mostly occurred in NEPC, as subsequently analyzed using cBioPortal. In contrast, this alteration regarding *GLS2* occurred primarily in lung cancer. Alterations in NEPC and lung cancer were found to occur largely due to amplifications and mutations, respectively. Moreover, the expression of combined *GLS-* and *GLS2*-centered- signature-gene set regulated clinical prognosis in certain cancers which further predict that the functional partners of *GLS* and *GLS2* contribute to significant CNAs and mutations and subsequently regulate the clinical outcomes in cancers.

We then determined the genes correlated with *GLS* and *GLS2* in certain types of cancers in which these glutaminase members are significantly upregulated using the R2 platform. For *GLS*, a large number of positively-correlated genes were found in breast, colon, liver, and head-and-neck cancer. Of those genes, 156 were common in all cancers. For *GLS2*, a considerably lower number of positively-correlated genes were associated with colon, head-and-neck, lung, and blood cancer, among which six genes were common in all cancers. In addition, we used the PANTHER and NOA web tools to determine the GO and pathways associated with these commonly correlated genes for each glutaminase member. Among the 10 pathways of each cluster, only two pathways were common in both *GLS*-centered positive and *GLS2*-centered positive clusters. Moreover, from a functional classification viewpoint, glutaminases were involved in varying biological processes, cellular components, and molecular functions. Taken together, the pathways and GO analyses suggest that, for the most part, *GLS* and *GLS2* perform different functions with respect to pathway regulation.

Next, based on their expression patterns, we evaluated the prognostic significance of glutaminases in various cancers using the R2, PrognoScan, Kaplan-Meier Plotter, and PROGgeneV2 databases. In general, high *GLS* expression resulted in poor survival in patients with breast, esophagus, head-and-neck, and blood cancer, and high survival in patients with bladder, kidney, and lung cancer. For *GLS2*, high expression resulted in poor survival in patients with colon, blood, ovarian, and thymoma cancer, and high survival in patients with brain, kidney, and skin cancer. Notably, whereas both high and low expression of *GLS* resulted in a poor prognosis in gastric, blood, and liver cancer, the same expression pattern was associated with low survival in bladder and pancreatic cancer. Some of these findings are in agreement with the previous studies [88,89,90,91]. In reference to colon cancer, in which *GLS2* expression is high, *GLS2* inhibitors were discovered, which could be used as a potential anti-cancer target [88]. *GLS2* was found to be an important predictor of survival for patients with liver cancer, where this gene is downregulated [89]. The *GLS* gene had a noticeable clinical significance in the assessment of recurrence, metastasis, and death in patients with breast cancer, in which this gene was upregulated [90]. *GLS* and *GLS2* inhibitors were discovered which were able to inhibit cell proliferation in various types of cancer, including breast, blood, and lung cancer [91]. Thus, we concluded that *GLS* plays an oncogenic role in a number of cancers, such as breast, esophagus, head-and-neck, blood, gastric, blood, and liver cancer. Likewise, *GLS2* shows oncogenic behavior in colon, blood, ovarian, thymoma, and bladder cancer. In contrast, whereas *GLS* demonstrated anti-oncogenic roles in bladder, kidney, and lung cancer, *GLS2* acted as a tumor suppressor in brain, kidney, pancreatic, and skin cancer. We also assessed the impact of *GLS*/*GLS2* co-expression on various types of cancer using multivariate survival analyses. Whereas the co-occurrence (high/high) of *GLS*/*GLS2* resulted in a poor prognosis in ovarian and thymoma cancer, the non-co-occurrence (low/low) of this gene pair resulted in low survival in patients with breast, brain, and colon cancer. In addition, the partial-occurrence (low/high) of glutaminases was associated with poor clinical outcomes in lung and gastric cancer. These results suggest that the co-expression of *GLS* and *GLS2* could also be utilized as a prognostic marker for patients with certain types of cancer.

To this end, we provide some insights into the integrated aspect of this multi-omics study. We investigated the expression, methylation, mutation, and copy number alteration patterns of *GLS* and *GLS2* genes and assessed their prognostic significance through a systematic data analysis, using publicly available expression and clinical data. This analysis demonstrated that the expression and methylation status of glutaminases collectively show biomarker significance for certain types of cancer, including esophageal, head-and-neck, kidney, and liver cancer. The biological basis of the biomarker significance possibly comes in part from the accumulated mutations and CNAs of the glutaminases along with their functional protein partners. Perhaps correlated genes also play a significant role to contribute to the altered biological functions through a diverse pathway regulation. These data suggest that glutaminases expression may be translated into clinical practice and that their co-expression may impact clinical outcomes. Moreover, the functional partners of *GLS* and *GLS2* also eventually regulate the survival patterns of patients with certain types of cancer. Our analysis shows that the upregulation of *GLS* with hypomethylation is positively correlated with poor prognosis in patients with breast, colon, and head-and-neck cancers. In these cancers, high expression of the functional partners of *GLS* is also significantly associated with low survival, which suggests that *GLS* and its functional partners converge in terms of their prognostic behavior and thus imposes a combined impact on the clinical outcomes in the certain cancers. Although our analysis demonstrated the association between expression and methylation stratus for *GLS2* in some cancers, the analysis does not establish an integrated survival pattern based on its expression, methylation, and functional partners together.

## 5. Conclusions

In this study, we used different online bioinformatics platforms and tools to systemically analyze the expression, methylation status, functional protein partners, correlated genes, and prognostic values of glutaminases in various human cancers. Our multiomics analysis revealed that *GLS* and *GLS2* play distinct roles in cancer development and differentially modulate the clinical outcomes of cancer. Whereas *GLS* could be targeted for cancer therapy in patients with breast, esophagus, head-and-neck, and blood cancer, *GLS2* could be utilized as a prognostic marker for colon, blood, ovarian, and thymoma cancer. The multivariate survival analysis of *GLS* and *GLS2* also predict the therapeutic targets for certain types of cancer, including ovarian, brain, and lung cancer. Moreover, expression of combined *GLS* and *GLS2*-centered-signature-gene set might regulate the clinical outcomes in certain cancer. Thus, these expressions of *GLS* and *GLS2* could be regulated through their promoter methylation. Moreover, functional protein partners and correlated genes may regulate the biological function of glutaminases which can subsequently affect clinical outcomes in cancers. In summary, the findings of this study provide some an insight into the molecular and clinical characteristics of different types of cancer and could thus be used to assist in transforming genomic knowledge into cancer therapy. Nevertheless, more theoretical, experimental, and clinical studies are required to validate the outcomes of this study, since the data mining-based analyses may appear in the form of overfitting and underfitting.

## Supplementary Materials

The supplementary materials are available online at https://www.mdpi.com/2077-0383/8/3/355/s1.
