# Supplementary material for: Multiomics Analysis Reveals that GLS and GLS2 Differentially Modulate the Clinical Outcomes of Cancer
Source: J Clin Med. 2019 Mar 13;8(3):355. doi: 10.3390/jcm8030355 (PMC6463114; doi:10.3390/jcm8030355)
Supplement: Supplementary file 1 [file jcm-08-00355-s001.pdf]

Multiomics Analysis Reveals that GLS and GLS2 Differentially Modulate the Clinical Outcomes of Cancer

Supplementary Table S1. GLS1 expression in various cancers (Oncomine database).

Order	Cancer	Cancer subtype	Fold change	p-value	No. of sample	Rank (%)	Reporter	Ref.
1		Superficial Bladder Cancer	-2.694	1.18E-09	157	7	221510_s_at	[1]
2	Brain and CNS	Anaplastic Oligoastrocytoma	6.348	4.90E-09	33	1	221510_s_at	[2]
		Glioblastoma	3.599	1.89E-05	84	7	221510_s_at	[3]
		Anaplastic Oligodendroglioma	3.191	1.32E-09	33	1	221510_s_at	[2]
		Glioblastoma	-7.148	1.29E-33	84	1	203159_at	[3]
		Brain Glioblastoma	-17.238	2.86E-24	557	1	203159_at	TCGA
3	Breast	Lobular Breast Carcinoma	3.861	1.31E-05	64	4	IMAGE:256895	Zhao Breast
		Ductal Breast Carcinoma in Situ	2.107	4.47E-04	66	4	g6002672_3p_a_at	Ma Breast 4
		Invasive Ductal Breast Carcinoma	3.088	5.11E-05	64	8	IMAGE:256895	Zhao Breast
4	Cervical	Cervical Squamous Cell Carcinoma	1.886	9.63E-05	41	6	203159_at	Zhai Cervix
		High Grade Cervical Squamous Intraepithelial Neoplasia	1.832	2.07E-04	41	2	203159_at	Zhai Cervix
		Cervical Cancer	2.846	2.34E-05	84	16	221510_s_at	Pyeon Multi-cancer
5	Colorectal	Colorectal Carcinoma	2.174	3.84E-11	105	2	221510_s_at	Skrzypczak Colorectal

		Colon Carcinoma	3.086	1.71E-06	40	7	223079_s_at	Skrzypczak Colorectal 2
		Colorectal Carcinoma	2.019	7.70E-09	82	7	221510_s_at	Hong Colorectal
6	Esophageal	Esophageal Adenocarcinoma	2.093	2.48E-05	48	3	IMAGE:1626076	Hao Esophagus
		Esophageal Squamous Cell Carcinoma	2.648	3.12E-06	34	5	221510_s_at	Hu Esophagus
		Esophageal Adenocarcinoma	3.391	0.004	24	7	221510_s_at	Kimchi Esophagus
		Esophageal Squamous Cell Carcinoma	1.715	6.40E-10	106	8	221510_s_at	Su Esophagus 2
7	Gastric	Diffuse Gastric Adenocarcinoma	2.146	7.73E-06	69	1	221510_s_at	DErrico Gastric
		Gastric Mixed Adenocarcinoma	3.646	5.35E-06	69	2	221510_s_at	DErrico Gastric
		Gastric Intestinal Type Adenocarcinoma	2.434	5.47E-10	69	4	221510_s_at	DErrico Gastric
8	Head and Nect	Nasopharyngeal Carcinoma	2.845	7.02E-08	41	1	223079_s_at	Sengupta Head-Neck
		Head and Neck Squamous Cell Carcinoma	2.906	1.37E-09	54	4	221510_s_at	Ginos Head-Neck
		Tongue Carcinoma	2.947	4.24E-05	84	7	221510_s_at	Pyeon Multi-cancer
9	Kidney	Renal Wilms Tumor	-15.398	1.13E-05	67	1	203159_at	Yusenko Renal
10	Leukemia	Chronic Lymphocytic Leukemia	3.042	8.18E-06	120	4	IMAGE:704179	Alizadeh Lymphoma
		Chronic Lymphocytic Leukemia	3.404	1.78E-06	102	3	AA279312 (1)	Rosenwald Multi-cancer
		Chronic Lymphocytic Leukemia	2.415	1.23E-06	111	3	34719_at	Haslinger Leukemia

11	Liver	Hepatocellular Carcinoma	2.349	5.28E-08	75	2	203159_at	Wurmbach Liver
		Hepatocellular Carcinoma	2.38	2.31E-07	43	4	203159_at	Roessler Liver
		Hepatocellular Carcinoma	1.78	3.44E-08	115	4	203159_at	Mas Liver
12	Lung	Lung Carcinoid Tumor	-8.648	1.42E-07	203	7	34719_at	Bhattacharjee Lung
		Small Cell Lung Carcinoma	-7.917	1.61E-05	203	4	34719_at	Bhattacharjee Lung
		Squamous Cell Lung Carcinoma	-6.244	2.18E-06	203	2	34719_at	Bhattacharjee Lung
13	Lymphoma	Anaplastic Large Cell Lymphoma	2.761	1.01E-10	60	1	203159_at	Piccaluga Lymphoma
		Follicular Lymphoma	2.656	4.98E-04	102	3	AA279312 (1)	Rosenwald Multi-cancer
14	Myeloma	Smoldering Myeloma	2.066	1.43E-06	78	7	221510_s_at	Zhan Myeloma 3
15	Other	Non-Familial Multiple Gland Neoplasia	2.127	2.37E-04	61	2	203157_s_at	Morrison Parathyroid
		Parathyroid Gland Adenoma	2.227	1.51E-04	61	4	203157_s_at	Morrison Parathyroid

Supplementary Table S2. GLS2 expression in various cancers (Oncomine database).

Order	Cancer	Cancer subtype	Fold change	p-value	No. of sample	Rank (%)	Reporter	Ref.
1	Bladder	Superficial Bladder Cancer	4.004	5.71E-16	157	4	205531_s_at	[1]
		Infiltrating Bladder Urothelial Carcinoma	2.526	0.012	93	8	AI268697	Blaveri Bladder 2
2	Brain and CNS	Brain Glioblastoma	-7.281	1.59E-09	557	7	205531_s_at	TCGA
		Glioblastoma	-8.875	2.95E-13	180	9	205531_s_at	[4]
		Anaplastic Oligodendroglioma	-7.234	3.17E-07	180	6	205531_s_at	[4]
5	Colorectal	Colon Mucinous Adenocarcinoma	6.605	1.41E-12	237	2	A_24_P326739	TCGA
		Colon Adenocarcinoma	5.195	1.82E-43	237	1	A_23_P36416	TCGA
		Colon Carcinoma	3.664	6.23E-10	40	2	205531_s_at	Skrzypczak Colorectal 2
		Colorectal Carcinoma	5.938	1.06E-09	82	6	1564706_s_at	Hong Colorectal
8	Head and Nect	Thyroid Gland Papillary Carcinoma	1.589	2.50E-04	18	2	205531_s_at	Vasko Thyroid
		Nasopharyngeal Carcinoma	2.028	2.36E-06	41	3	205531_s_at	Sengupta Head-Neck
9	Leukemia	T-Cell Acute Lymphoblastic Leukemia	2.44	6.64E-05	127	10	IMAGE:1911706	Andersson Leukemia
10	Liver	Hepatocellular Carcinoma	-15.717	7.80E-12	43	1	205531_s_at	Roessler Liver
		Hepatocellular Carcinoma	-10.714	3.81E-73	445	1	205531_s_at	Roessler Liver 2

11	Lung	Lung Adenocarcinoma	4.475	9.69E-07	39	3	35711_at	Stearman Lung
		Lung Carcinoid Tumor	3.324	3.00E-03	203	8	35711_at	Bhattacharjee Lung
		Lung Adenocarcinoma	2.283	9.71E-05	66	7	205531_s_at	Su Lung
12	Ovarian	Ovarian Serous Adenocarcinoma	5.611	4.51E-05	53	8	A_23_P36416	Yoshihara Ovarian
13	Pancreatic	Pancreatic Adenocarcinoma	-19.388	1.66E-06	36	1	IMAGE:1911706	Iacobuzio-Donahue Pancreas 2

Supplementary Table S3. The association of GLS1 expression and the survival in cancer patients (Prognoscan database).

Order	CANCER TYPE	ENDPOINT	N	COX P-VALUE	HR [95% CI^{low} - CI^{upp}]	ARRAY TYPE	DATASET	PROBE ID
1	Brain cancer	Overall Survival	77	2.15E-02	0.59 [0.38 - 0.93]	HG-U133A	GSE4271-GPL96	203159_at
		Overall Survival	74	2.97E-02	0.42 [0.19 - 0.92]	HG-U133A	GSE4412-GPL96	221510_s_at
2	Breast cancer	Distant Metastasis Free Survival	200	4.30E-05	0.40 [0.25 - 0.62]	HG-U133A	GSE11121	203157_s_at
		Relapse Free Survival	204	1.22E-03	1.45 [1.16 - 1.82]	HG-U133_Plus_2	GSE12276	221510_s_at
		Overall Survival	155	1.31E-03	1.53 [1.18 - 1.99]	MLRG Human 21K V12.0	GSE9893	5827
		Distant Metastasis Free Survival	286	2.07E-02	0.75 [0.58 - 0.96]	HG-U133A	GSE2034	203157_s_at
		Disease Specific Survival	236	3.51E-02	0.73 [0.54 - 0.98]	HG-U133B	GSE3494-GPL97	241034_at
		Distant Metastasis Free Survival	136	4.86E-02	0.56 [0.32 - 1.00]	HG-U133A	GSE12093	203157_s_at
3	Blood cancer	Overall Survival	163	2.21E-02	1.56 [1.07 - 2.29]	HG-U133B	GSE12417-GPL97	223079_s_at
4	Colorectal cancer	Disease Specific Survival	49	3.43E-03	0.03 [0.00 - 0.30]	HG-U133_Plus_2	GSE17537	223080_at
		Disease Free Survival	55	1.00E-02	0.08 [0.01 - 0.54]	HG-U133_Plus_2	GSE17537	223080_at
		Overall Survival	55	1.76E-02	0.12 [0.02 - 0.69]	HG-U133_Plus_2	GSE17537	223080_at
5	Eye cancer	Distant Metastasis Free Survival	63	2.26E-03	0.62 [0.45 - 0.84]	HG-U133_Plus_2	GSE22138	221510_s_at
6	Lung cancer	Overall Survival	204	3.70E-05	0.51 [0.37 - 0.71]	HG-U133_Plus_2	GSE31210	241034_at

		Relapse Free Survival	204	1.18E-03	0.66 [0.52 - 0.85]	HG-U133_Plus_2	GSE31210	241034_at
		Overall Survival	129	1.96E-02	0.50 [0.28 - 0.90]	HG-U133A	GSE4573	203157_s_at
		Relapse Free Survival	138	3.93E-02	0.00 [0.00 - 0.59]	HG-U133_Plus_2	GSE8894	211414_at
		Overall Survival	82	4.97E-02	0.44 [0.20 - 1.00]	HG-U133A	jacob-00182-CANDF	203158_s_at
7	Ovarian cancer	Overall Survival	278	7.08E-04	1.59 [1.21 - 2.07]	HG-U133_Plus_2	GSE9891	221510_s_at
		Overall Survival	185	5.44E-03	1.52 [1.13 - 2.04]	HG-U133_Plus_2	GSE26712	221510_s_at
		Disease Free Survival	185	6.72E-03	1.46 [1.11 - 1.92]	HG-U133_Plus_2	GSE26712	221510_s_at
		Overall Survival	133	2.08E-02	14.39 [1.50 - 138.05]	HG-U133A	DUKE-OC	211414_at
8	Soft tissue cancer	Distant Recurrence Free Survival	140	2.38E-02	2.35 [1.12 - 4.93]	HG-U133A	GSE30929	221510_s_at

Supplementary Table S4. The association of GLS2 expression and the survival in cancer patients (Prognoscan database).

Order	CANCER TYPE	ENDPOINT	N	COX P-VALUE	HR [95% CI^{low} - CI^{upp}]	ARRAY TYPE	DATASET	PROBE ID
1	Breast cancer	Relapse Free Survival	204	1.73E-04	0.80 [0.72 - 0.90]	HG-U133_Plus_2	GSE12276	1564707_x_at
		Distant Metastasis Free Survival	87	7.89E-04	26.88 [3.93 - 183.73]	HG-U133_Plus_2	GSE6532-GPL570	1564707_x_at
		Relapse Free Survival	87	7.89E-04	26.88 [3.93 - 183.73]	HG-U133_Plus_2	GSE6532-GPL570	1564707_x_at
		Disease Free Survival	249	2.79E-02	0.71 [0.53 - 0.96]	HG-U133A	GSE4922-GPL96	205531_s_at
		Disease Specific Survival	117	4.32E-02	2.26 [1.03 - 4.98]	HG-U133A	E-TABM-158	205531_s_at
2	Colorectal cancer	Disease Free Survival	55	2.46E-02	28.83 [1.54 - 540.03]	HG-U133_Plus_2	GSE17537	1564707_x_at
		Disease Specific Survival	177	2.90E-02	1.90 [1.07 - 3.38]	HG-U133_Plus_2	GSE17536	1564706_s_at
		Overall Survival	55	4.33E-02	26.88 [1.10 - 654.41]	HG-U133_Plus_2	GSE17537	1564707_x_at
3	Eye cancer	Distant Metastasis Free Survival	63	3.05E-02	0.00 [0.00 - 0.15]	HG-U133_Plus_2	GSE22138	1564706_s_at
4	Lung cancer	Overall Survival	204	1.85E-03	0.67 [0.52 - 0.86]	HG-U133_Plus_2	GSE31210	1564706_s_at
		Overall Survival	178	5.53E-03	0.70 [0.54 - 0.90]	HG-U133A	jacob-00182-UM	205531_s_at
		Relapse Free Survival	204	1.39E-02	0.77 [0.63 - 0.95]	HG-U133_Plus_2	GSE31210	205531_s_at
5	Ovarian cancer	Overall Survival	81	2.58E-03	5.83 [1.85 - 18.38]	G4100A	GSE8841	6698
		Overall Survival	80	3.02E-02	0.65 [0.44 - 0.96]	HG-U133A	GSE14764	205531_s_at

		Overall Survival	110	3.19E-02	0.66 [0.46 - 0.97]	G4112A	GSE17260	A_24_P326739
6	Skin cancer	Overall Survival	38	4.76E-02	0.66 [0.43 - 1.00]	HG-U133_Plus_2	GSE19234	1564707_x_at

Supplementary Table S5. Gene ontology (GO) of positively correlated genes of GLS1 (Network Ontology Analysis database).

GO: term	p-value	Corrected p-value	Term name
**Biological Processes**			
GO:0044260	1.50E-06	0.0015	cellular macromolecule metabolic process
GO:0016070	2.40E-06	0.0024	RNA metabolic process
GO:0043170	1.90E-05	0.0193	macromolecule metabolic process
GO:0090304	2.30E-05	0.0236	nucleic acid metabolic process
GO:0006139	4.40E-05	0.0446	nucleobase, nucleoside, nucleotide and nucleic acid metabolic process
GO:0006396	2.00E-04	0.204	RNA processing
GO:0034641	2.60E-04	0.2588	cellular nitrogen compound metabolic process
GO:0043412	2.80E-04	0.2847	macromolecule modification
GO:0023034	4.50E-04	0.4508	intracellular signaling pathway
GO:0006807	6.60E-04	0.6548	nitrogen compound metabolic process
**Cellular Components**			
GO:0044428	1.40E-05	0.0026	nuclear part
GO:0005634	2.60E-05	0.0048	nucleus
GO:0044446	1.10E-04	0.0203	intracellular organelle part
GO:0044422	1.60E-04	0.0298	organelle part
GO:0044424	1.80E-04	0.0335	intracellular part
GO:0043229	3.60E-04	0.0667	intracellular organelle
GO:0043226	3.80E-04	0.0712	organelle
GO:0043231	6.50E-04	0.1205	intracellular membrane-bounded organelle
GO:0043227	6.60E-04	0.1216	membrane-bounded organelle
GO:0044451	8.30E-04	0.1544	nucleoplasm part

Molecular Functions

GO:0032553	1.70E-04	0.0494	ribonucleotide binding
GO:0032555	1.70E-04	0.0494	purine ribonucleotide binding
GO:0032559	2.30E-04	0.0688	adenyl ribonucleotide binding
GO:0017076	3.50E-04	0.103	purine nucleotide binding
GO:0000166	3.90E-04	0.1155	nucleotide binding
GO:0005524	4.80E-04	0.1403	ATP binding
GO:0030554	5.10E-04	0.1498	adenyl nucleotide binding
GO:0005488	5.50E-04	0.1609	binding
GO:0001883	7.10E-04	0.2063	purine nucleoside binding
GO:0001882	7.70E-04	0.2249	nucleoside binding

Supplementary Table S6. Gene ontology (GO) of negatively correlated genes of GLS1 (Network Ontology Analysis database).

GO: term	p-value	Corrected p-value	Term name
**Biological Processes**			
GO:0008152	1.70E-06	7.00E-04	metabolic process
GO:0044237	5.60E-06	0.0023	cellular metabolic process
GO:0051436	3.00E-05	0.0125	negative regulation of ubiquitin-protein ligase activity involved in mitotic cell cycle
GO:0031145	3.20E-05	0.0134	anaphase-promoting complex-dependent proteasomal ubiquitin-dependent protein catabolic process
GO:0051437	3.60E-05	0.0151	positive regulation of ubiquitin-protein ligase activity involved in mitotic cell cycle
GO:0051352	3.90E-05	0.0161	negative regulation of ligase activity
GO:0051444	3.90E-05	0.0161	negative regulation of ubiquitin-protein ligase activity
GO:0051439	4.40E-05	0.0181	regulation of ubiquitin-protein ligase activity involved in mitotic cell cycle
GO:0051443	4.90E-05	0.0203	positive regulation of ubiquitin-protein ligase activity
GO:0051351	5.80E-05	0.024	positive regulation of ligase activity
GO:0005739	1.10E-08	1.10E-06	mitochondrion
**Cellular Components**			
GO:0044429	1.30E-05	0.0012	mitochondrial part

GO:0008537	2.70E-05	0.0025	proteasome activator complex
GO:0043231	2.70E-04	0.0254	intracellular membrane-bounded organelle
GO:0043227	2.70E-04	0.0256	membrane-bounded organelle
GO:0044444	8.00E-04	0.0753	cytoplasmic part
GO:0005743	0.001	0.0959	mitochondrial inner membrane
GO:0070469	0.0011	0.104	respiratory chain
GO:0005759	0.0011	0.1042	mitochondrial matrix
GO:0019866	0.0015	0.1453	organelle inner membrane
GO:0016491	3.50E-05	0.0061	oxidoreductase activity
**Molecular Functions**			
GO:0016681	2.10E-04	0.0367	oxidoreductase activity, acting on diphenols and related substances as donors, cytochrome as acceptor
GO:0008121	2.10E-04	0.0367	ubiquinol-cytochrome-c reductase activity
GO:0003824	2.50E-04	0.0439	catalytic activity
GO:0016679	2.60E-04	0.0458	oxidoreductase activity, acting on diphenols and related substances as donors
GO:0015078	0.0015	0.2693	hydrogen ion transmembrane transporter activity
GO:0015140	0.0024	0.4279	malate transmembrane transporter activity
GO:0015367	0.0024	0.4279	oxoglutarate:malate antiporter activity
GO:0016034	0.0024	0.4279	maleylacetoacetate isomerase activity
GO:0016730	0.0024	0.4279	oxidoreductase activity, acting on iron-sulfur proteins as donors

Supplementary Table S7. Gene ontology (GO) of positively correlated genes of GLS2 (Network Ontology Analysis database).

GO: term	p-value	Corrected p-value	Term name
**Biological Processes**			
GO:0009083	1.20E-07	5.10E-05	branched chain family amino acid catabolic process
GO:0009081	5.90E-07	2.40E-04	branched chain family amino acid metabolic process
GO:0009063	1.90E-04	0.0791	cellular amino acid catabolic process
GO:0055114	2.70E-04	0.1114	oxidation reduction
GO:0009310	3.40E-04	0.1388	amine catabolic process
GO:0016054	0.0014	0.5716	organic acid catabolic process
GO:0046395	0.0014	0.5716	carboxylic acid catabolic process
GO:0006520	0.0024	0.9926	cellular amino acid metabolic process
GO:0045449	0.0039	1	regulation of transcription
GO:0019484	0.0041	1	beta-alanine catabolic process
**Cellular Components**			
GO:0044429	1.60E-07	1.40E-05	mitochondrial part
GO:0005739	4.20E-07	3.80E-05	mitochondrion
GO:0005759	2.70E-06	2.40E-04	mitochondrial matrix
GO:0043229	6.90E-06	6.20E-04	intracellular organelle
GO:0043226	7.30E-06	6.60E-04	organelle
GO:0043231	8.10E-06	7.30E-04	intracellular membrane-bounded organelle

GO:0043227	8.20E-06	7.30E-04	membrane-bounded organelle
GO:0044424	1.40E-05	0.0012	intracellular part
GO:0005622	7.90E-05	0.0071	intracellular
GO:0034464	3.40E-04	0.0311	BBSome
**Molecular Functions**			
GO:0003995	4.30E-05	0.0088	acyl-CoA dehydrogenase activity
GO:0016627	1.00E-04	0.0208	oxidoreductase activity, acting on the CH-CH group of donors
GO:0050662	2.10E-04	0.0429	coenzyme binding
GO:0008270	3.00E-04	0.0629	zinc ion binding
GO:0003677	3.70E-04	0.0766	DNA binding
GO:0016903	4.80E-04	0.0998	oxidoreductase activity, acting on the aldehyde or oxo group of donors
GO:0046872	5.70E-04	0.1166	metal ion binding
GO:0046914	6.50E-04	0.1337	transition metal ion binding
GO:0043169	6.90E-04	0.141	cation binding
GO:0043167	7.20E-04	0.1478	ion binding

Supplementary Table S8. Gene ontology (GO) of negatively correlated genes of GLS2 (Network Ontology Analysis database).

GO: term	p-value	Corrected p-value	Term name
**Biological Processes**			
GO:0006928	2.50E-10	1.50E-07	cellular component movement
GO:0008064	1.70E-08	1.10E-05	regulation of actin polymerization or depolymerization
GO:0030832	2.10E-08	1.30E-05	regulation of actin filament length
GO:0032535	1.30E-07	8.30E-05	regulation of cellular component size
GO:0032956	2.20E-07	1.40E-04	regulation of actin cytoskeleton organization
GO:0032970	2.70E-07	1.70E-04	regulation of actin filament-based process
GO:0030833	4.50E-07	2.80E-04	regulation of actin filament polymerization
GO:0032271	1.00E-06	6.50E-04	regulation of protein polymerization
GO:0090066	1.10E-06	7.00E-04	regulation of anatomical structure size
GO:0051128	1.60E-06	0.001	regulation of cellular component organization
**Cellular Components**			
GO:0005885	1.30E-09	1.90E-07	Arp2/3 protein complex
GO:0005856	1.80E-08	2.70E-06	cytoskeleton
GO:0030863	1.10E-06	1.60E-04	cortical cytoskeleton
GO:0015629	1.20E-06	1.80E-04	actin cytoskeleton
GO:0005737	4.50E-06	6.60E-04	cytoplasm
GO:0044448	3.30E-05	0.0049	cell cortex part
GO:0002102	9.50E-05	0.014	podosome
GO:0043228	1.00E-04	0.0151	non-membrane-bounded organelle
GO:0043232	1.00E-04	0.0151	intracellular non-membrane-bounded organelle
GO:0030055	1.00E-04	0.0154	cell-substrate junction
**Molecular Functions**			

GO:0008092	8.50E-10	1.30E-07	cytoskeletal protein binding
GO:0003779	1.30E-09	2.10E-07	actin binding
GO:0005515	1.00E-04	0.0169	protein binding
GO:0005200	0.0011	0.1843	structural constituent of cytoskeleton
GO:0008418	0.0027	0.4492	protein N-terminal asparagine amidohydrolase activity
GO:0009032	0.0027	0.4492	thymidine phosphorylase activity
GO:0016936	0.0055	0.8973	galactoside binding
GO:0001847	0.0055	0.8973	opsonin receptor activity
GO:0030395	0.0055	0.8973	lactose binding
GO:0032050	0.0055	0.8973	clathrin heavy chain binding

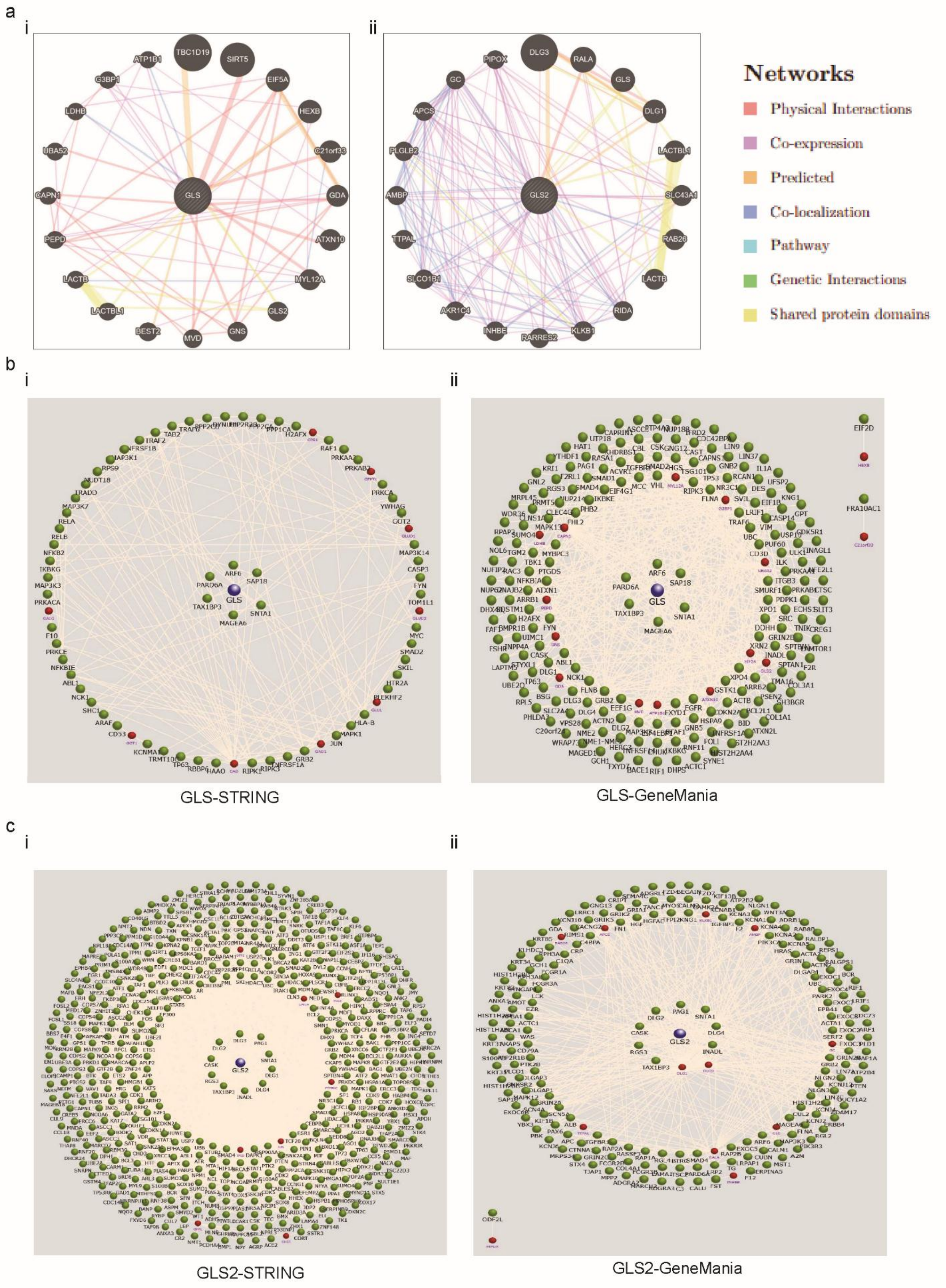
a
i
ii
TBC1D19
SIRT5
ATP1B1
G3BP1
EIF5A
LDHB
HEXB
UBA52
C21orf33
CAPN1
GLS
GDA
PEPD
ATXN10
LACTB
MYL12A
LACTBL1
GLS2
BEST2
MVD
GNS
DLG3
PIPOX
RALA
GC
GLS
DLG1
APCS
PLGLB2
LACTBL1
AMBP
GLS2
SLC43A1
TTPAL
RAB26
SLCO1B1
LACTB
AKR1C4
RIDA
INHBE
RARRES2
KLKB1
Networks
Physical Interactions
Co-expression
Predicted
Co-localization
Pathway
Genetic Interactions
Shared protein domains
b
i
ii
GLS-STRING
GLS-GeneMania
c
i
ii
GLS2-STRING
GLS2-GeneMania

Supplementary Figure S1. Identification of known and predicted structural protein partners essential for GLS and GLS2 (GeneMania and Funrich). (**a**) Interacting nodes are displayed in circles using Genemania web. Predicted functional partners of GLS (**i**) and GLS2 (**ii**) are shown based upon considering co-expression, co-localization, genetic interactions, pathway, physical interactions, predicted, shared protein domains. (**b**) Interacting nodes for GLS are displayed in circles using FunRich program. Predicted functional partners of GLS retrieved from STRING were further analyzed using FunRich program (**i**). Predicted functional partners of GLS retrieved from GeneMania were further analyzed using FunRich program (**ii**). (**c**) Interacting nodes for GLS2 are displayed in circles using FunRich program. Predicted functional partners of GLS2 retrieved from STRING were further analyzed using FunRich program (**i**). Predicted functional partners of GLS2 retrieved from GeneMania were further analyzed using FunRich program (**ii**).

References

1. Sanchez-Carbayo, M.; Socci, N.D.; Lozano, J.; Saint, F.; Cordon-Cardo, C. Defining molecular profiles of poor outcome in patients with invasive bladder cancer using oligonucleotide microarrays. *J Clin Oncol* **2006**, *24*, 778-789, doi:10.1200/Jco.2005.03.2375.
2. French, P.J.; Swagemakers, S.M.A.; Nagel, J.H.A.; Kouwenhoven, M.C.M.; Brouwer, E.; van der Spek, P.; Luider, T.M.; Kros, J.M.; van den Bent, M.J.; Smitt, P.A.S. Gene expression profiles associated with treatment response in oligodendrogliomas. *Cancer Res* **2005**, *65*, 11335-11344, doi:10.1158/0008-5472.CAN-05-1886.
3. Murat, A.; Migliavacca, E.; Gorlia, T.; Lambiv, W.L.; Shay, T.; Hamou, M.F.; de Tribolet, N.; Regli, L.; Wick, W.; Kouwenhoven, M.C.M., et al. Stem cell-related "Self-Renewal" signature and high epidermal growth factor receptor expression associated with resistance to concomitant chemoradiotherapy in glioblastoma. *J Clin Oncol* **2008**, *26*, 3015-3024, doi:10.1200/Jco.2007.15.7164.
4. Sun, L.X.; Hui, A.M.; Su, Q.; Vortmeyer, A.; Kotliarov, Y.; Pastorino, S.; Passaniti, A.; Menon, J.; Walling, J.; Bailey, R., et al. Neuronal and glioma-derived stem cell factor induces angiogenesis within the brain. *Cancer Cell* **2006**, *9*, 287-300, doi:10.1016/j.ccr.2006.03.003.
